# Bio- and Fossil-Based Polymeric Blends and Nanocomposites for Packaging: Structure–Property Relationship

**DOI:** 10.3390/ma12030471

**Published:** 2019-02-03

**Authors:** Francesca Luzi, Luigi Torre, José Maria Kenny, Debora Puglia

**Affiliations:** Civil and Environmental Engineering Department, University of Perugia, UdR INSTM, Strada di Pentima 4, 05100 Terni, Italy; francesca.luzi@unipg.it (F.L.); luigi.torre@unipg.it (L.T.); jose.kenny@unipg.it (J.M.K.)

**Keywords:** bio-based, fossil, hybrids, blends, packaging

## Abstract

In the present review, the possibilities for blending of commodities and bio-based and/or biodegradable polymers for packaging purposes has been considered, limiting the analysis to this class of materials without considering blends where both components have a bio-based composition or origin. The production of blends with synthetic polymeric materials is among the strategies to modulate the main characteristics of biodegradable polymeric materials, altering disintegrability rates and decreasing the final cost of different products. Special emphasis has been given to blends functional behavior in the frame of packaging application (compostability, gas/water/light barrier properties, migration, antioxidant performance). In addition, to better analyze the presence of nanosized ingredients on the overall behavior of a nanocomposite system composed of synthetic polymers, combined with biodegradable and/or bio-based plastics, the nature and effect of the inclusion of bio-based nanofillers has been investigated.

## 1. Introduction

In the last decades, after the signing of the environmental treaty on the 11th December 1997, that became law on the 16th February 2005, the environmental issue related to greenhouse gas emissions and climate changes was definitively raised and made one of the most important worldwide concerns, particularly in the face of toxic waste, pollution, contamination, exhaustion of natural resources, and environmental deterioration. As a result, different studies have been followed at diverse stages to develop different and strategic alternatives [1]. A serious alarm is the field of packaging, which, every year, causes enormous quantities of petroleum-based wastes that are stored in particular areas around the planet (26% of the plastic manufacture volume has been applied in the packaging sector) [2], determining enormous negative effects and high recycling costs [3]. In the future years, especially in 2030 and 2050, it was estimated that the quantity of plastic wastes due to the packaging sector will be grown by two-fold and three-fold, respectively [4]. Notwithstanding the environmental impacts, plastic materials are extremely useful in the packaging sector, due to positive and synergic combination of main characteristics, such as transparency, strength ability, flexibility, thermal performance, permeability, and simple sterilization methods, all of which making them appropriate for the food packaging sector. Hitherto, petroleum-based polymers (i.e., ethylene vinyl alcohol (EVOH), polypropylene (PP), polyethylene (PE), polyurethane (PU), poly (ethylene terephthalate) (PET), polystyrene (PS), expanded polystyrene, polyamides (PA), and poly (vinyl chloride) (PVC)) have led in the packaging function, for excellent mechanical and physical characteristics. However, according to results from plastics recycling and recovery data obtained from European associations in 2014, only 39.5% of post-consumer plastic waste is going to be re-used, while 38.6% of post-consumer plastic waste is considered for energy recovery [5]. With the intention of minimizing the environmental impacts induced by post-consumer plastic waste, bio-based polymers should be selected to realize short-lifespan devices. Environmentally friendly systems are appropriate solutions to realize disposable systems [6,7]; on the other hand, green polymeric systems are only used for some specific applications, due to their limiting characteristics, such as high cost and scarce mechanical and thermomechanical properties with respect of traditional commodity polymers. Developing green and eco-friendly polymeric blends with acceptable characteristics can overcome these limitations, even if it has been recently demonstrated that biodegradable plastic blends need both accurate cautious postconsumer organization and additional design to consent fast biodegradation in numerous environment conditions (as their release into the environment can determine plastic pollution) [8].

Preparation of blends with synthetic polymers [9,10,11] is among the options to enhance some characteristics of biodegradable polymers, changing degradation rates and modulating the cost of the obtained materials; polymer blends, particularly olefins with biodegradable polymers, are gaining popularity as an approach for degradable packaging plastics, since the partial loss of form and bulk during disintegration may be sufficient to decrease the volume in landfill [12]. This blending approach began in the 1970s at the U.S.D.A. with Otey [13], who studied and investigated blends based on starch and ethylene/acrylic acid copolymers and still now starch, being cheap, continues to be an attractive substitute to realize systems for the packaging sector [14]. In addition, to expand the spectrum of sustainability incorporating resources and practices that move a step closer toward sustainability [15], growing the renewable amount or lessening the overall weight of petroleum-based plastics have been considered as suitable options. Today’s sustainable plastics are not automatically biodegradable and even contain polyolefins made from renewable feedstocks [16,17].

In the present review, the blending of commodities and bio-based and/or biodegradable polymers will be taken into account (limiting the study to this class of materials and not considering blends where both components have a bio-based composition or origin), and special emphasis will be given to their functional behavior in terms of packaging application (compostability, gas/water/light barrier properties, migration, antioxidant performance). In addition, to better analyze the effect of green nanosized ingredients on the overall behavior of systems composed of synthetic polymers, combined with biodegradable and/or bio-based plastics, the effect of the inclusion of bio-based nanofillers has been investigated.

## 2. Bio-Based Nanofillers in the Packaging Sector

Recently, the growth of nanotechnology approaches and strategies has made their use become of interest in several sectors. Automotive, aerospace, biomedical, and packaging sectors have adopted and largely investigated the use of nanotechnology applications, as valid strategies to modulate and improve the characteristic main properties required in specific sectors [18]. Nanotechnology allows the realization of new systems to enhance material performances; of particular note is the recent development of nanocomposite systems that permitted the advancement of new polymeric-based formulations, with enhanced structural and functional properties (thermal, electrical, mechanical, and numerous other characteristics, in respect to the neat polymers [19,20,21,22,23]. Different nanocomposite-based systems have been realized by combining different polymers (petroleum-based and biodegradable/bio-based), and fillers at the nanoscale level. The nanofillers show strong reinforcing effects, several works have also analyzed their positive behavior in terms of barrier and mechanical properties, characteristics of essential importance in packaging and food packaging applications [24,25].

In this review, the current status of nanotechnology in packaging and also in food packaging systems are briefly reviewed and summarized. Nanofillers can be extracted from organic or inorganic sources; here the authors focused their attention to describe the main characteristics of nanofillers extracted from bio-based/natural sources (plant and animal origin and nanofillers from proteins) applied in the packaging sector. An explanation of different nanofillers, with an emphasis on the functionality, synthesis, characteristics, and structure is included.

### 2.1. Nanofillers from Polysaccharides—Plant Origin

In literature, different works have proposed the study of extraction and analysis of nanofillers from polysaccharides with a plant origin: cellulose nanofibers/nanocrystals, lignin, and starch nanoparticles. The lignocellulosic source is one of the most copious renewable materials existing in the world; these materials are natural, eco-friendly, sustainable, biodegradable, and considered as low-cost materials, with advantageous properties and with a significant value for packaging and industrial sectors. In comparison with petroleum-based natural sources, some interesting advantages are found: (i) low density and low cost, (ii) high variety, (iii) specific modulus and strength, (iv) reactive surfaces that can be changed and functionalized by a large variety of reactive chemical groups, (v) high applicability in nanocomposites, (vi) high recyclability in respect to inorganic fillers [25,26]. Lignocellulosic materials are generally composed by cellulose (40–50 wt %), hemicellulose (20–30 wt %), and lignin (about 10–25 wt %), and the quantities of the different components can be different according to the native lignocellulosic origin source [27]. 

#### 2.1.1. Cellulose Nanofibers/Nanocrystals 

**Figure 1:**
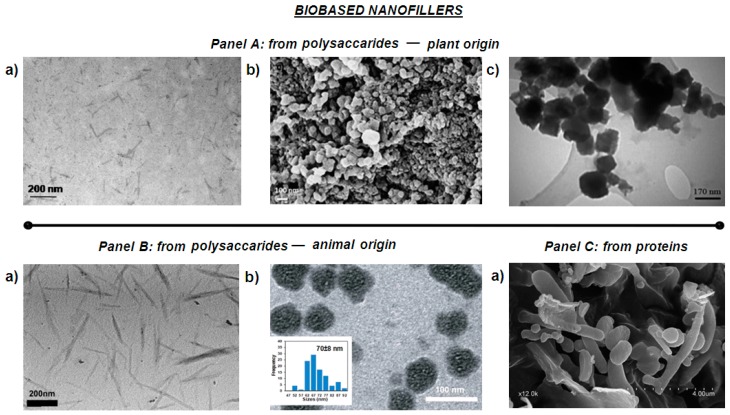
Morphological characterization of bio-based nanofillers. Panel A: Polysaccharides—plant origin: (**a**) Transmission Electron Microscopy (TEM) image of Cellulose Nanocrystals CNC [27]; (**b**) Field Emission Scanning Electron Microscopy (FESEM) image of lignin nanoparticles [34]; (**c**) TEM image of starch nanoparticles [35]. Panel B: Nanofillers from Polysaccharides—animal origin: (**a**) TEM image of Chitin nanocrystals [36], (**b**) TEM image of modified chitosan nanoparticles (CSNP) by poly (ethylene glycol) methyl ether methacrylate (PEGMA) (PEGMA-graft-CSNP) [37]. Panel C: From proteins: (**a**) Scanning Electron Microscopy (SEM) image of nanokeratin [38].

Cellulose is the natural polymer largely diffused on Earth, with excellent biocompatibility, good chemical and thermal stability, and high hydrophilicity. These attractive characteristics have determined cellulose as an interesting material for different applications in packaging and in biomedical applications. The cellulosic nanofillers are categorized on the basis of preparation methods considered for their extraction from native cellulose; they can be found as bacterial cellulose (BC) synthesized through microorganisms, microfibrillated cellulose (MFC) or nanofibrillated cellulose (NFC), or cellulose nanocrystals/nanocrystalline cellulose, also named cellulose nanowhiskers, (CNC). MCF is pulled out by means of a mechanical retting/disintegration method, starting from a variety of cellulosic extracts, including wood and non-wood fibers [28], consequently obtaining cellulose microfibrils with a three-dimensional network, the obtained structures showed higher surface area than original cellulosic fibers or from cellulosic powder. This effect influences a number of extremely interesting characteristics, such as an exceptionally high-water holding capability and the capacity to realize a configuration with strong gels at low concentrations. Although microfibrillated cellulose is not soluble in water, it can show several characteristics of water-soluble cellulose derivatives. Simultaneously, it has some advantages, such as stability over the whole pH range, at elevated temperatures and at elevated salt concentrations [29]. MFC show lengths in micrometers and diameters in nanometers, characterizing them as long and thin reinforcements. This elevated aspect ratio characterizes the material high strength as functional in several applications, such as the reinforcement phase for composites and films, and as an agent to modulate the barrier performance. Chemically extracted CNC are characterized by acicular structure and rigid rod-like particles, monocrystalline domains of 100 to hundreds of nanometers in length, and 1–100 nm in diameter (Figure 1, Panel A a)) [30,31]; morphology and crystallinity degree depend, fundamentally, on the native source and the different parameters used for the extraction process [27]. The extraordinary mechanical characteristics (Young’s modulus is higher than glass fibers and comparable to Kevlar (60–125 GPa)) give to cellulose crystal the role of a perfect filler material for the preparation of polymer composites. CNCs exhibit enormous applications in the biomedical sector and in bio-based material science [32,33].

Cellulose nanocrystals were largely used and applied to realize nanocomposites with modulated properties in respect to the neat matrices. Several studies, reported in literature, analyzed their effect in biodegradable matrices and polymeric blends, even in the presence of natural active ingredients and antimicrobial nanoparticles [24,39,40,41]. Cellulose nanocrystals are also used as the reinforcement phase in conventional matrices [42,43,44,45], by also providing, at the same time, an enhancement in terms of barrier properties.

#### 2.1.2. Lignin Nanoparticles

Lignin is the second most abundant aromatic polymeric material on earth. It is a cross-linked macromolecule in a three-dimensional shape, composed by three typologies of alternative phenols, yielding numerous functional groups and linkages, with a mutable chemistry as a consequence of its native source [34,46,47]. Lignin is an efficient phase to be included in polymers. Works on the applicability of micro-lignin in thermally processable plastic matrices, elastomers, and thermoset-based systems have been recently investigated [48,49,50]. Figure 1, Panel A, (b) shows the typical morphological aspect of lignin nanoparticles (LNP). LNP diameters are distributed in the range from 30 to 90 nm [47]. Recently, LNPs from a variety of native origins were synthesized/extracted by applying physicochemical procedures [34,51,52]. Furthermore, the lignin tendency to self-aggregate shows some disadvantages in terms of their dispersion in thermoplastic, thermoset, or elastomer-based systems. Therefore, many strategies have been considered and attempted in order to improve the dispersion of lignin particles into bio-based polymers [51].

#### 2.1.3. Starch Nanoparticles

Starch is composed of amylose and amylopectin. The amylose is a linear and long molecule built up of 1,4-linked β-D-glucose, although in the amylopectin chains the glucose monomers are linked through α-1,6-linkages, determining an extremely branched arrangement. Therefore, the molecular structure of amylose is simpler than amylopectin, showing a linear structure with few α-1,6-branches [53]. Amylopectin generally is the main component of starch, composed by short chains and a high number of α-1,6-branches (5% of the molecule) [53], while amylose, in general, is randomly arranged among the amylopectin molecules in the amorphous regions. Amylose plays an important part on the structure of the amylopectin in the crystalline lamellae by cross-linking the two polysaccharides [54].

Starch consists in granules, with diameters ranging from 2 to 100 μm. In relation to their native extraction starch, they have different characteristics properties, different chemical composition, different shape and size [21], that can be small (3.1–3.7 nm) and large (15–19 nm), and disc-shaped and/or spheroidal [55]. 

The purification of starch granules with appropriate chemical treatments allow one to obtain nanoparticles (SNP). In general, many different approaches can be selected and applied to starch with the intention to obtain granules, while their conversion in NPs is usually carried out by applying acid hydrolysis. The structural differences in starch granule sizes affects the outcome of the process in terms of starch purification, characteristics, and nanocrystal yield [19,56]. The acidic hydrolysis process permits one to obtain crystals with a spherical shape and dimensions ranging 20–50 nm [19,56].

### 2.2. Nanofillers from Polysaccharides—Animal Origin

Chitin and chitosan nanofillers can be isolated from α-chitin powder extracted from lobster wastes. The extraction of chitin and chitosan represents the possibility to revalorize the oceanic biomass and the revalorization of food extracts from the fishing industry. Lobster wastes are eco-friendly, renewable, sustainable, low cost, and biodegradable, with advantageous properties and with a significant value for packaging and biomedical applications.

#### 2.2.1. Chitin Nanoparticles/Nanofibers

Chitin is one of the most copious natural polymers obtained from shellfish waste (exoskeleton/shells), and it is used in combination with biopolymers to realize nanocomposites with modulated properties, in respect to the neat matrices, principally for food packaging and biomedical applications. Chitin is extracted from cuticles of insects and exoskeleton of arthropods at the micro/nanoscale (length: 200–300 nm; diameter: 10–20 nm) [57]. Different treatments can be used to extract crystalline chitin in nanosized fibrils, the different methods influenced also the dimension and the morphology of extracted materials [58,59,60]. The isolation of these nanosized structures can be performed by: (i) mechanical treatments/disintegration [57,61], resulting in chitin nanofibers (CHNF) and fibrils with high aspect ratio; (ii) acidic treatments [60,61,62], resulting in chitin nanocrystals (CHNC), with higher crystallinity degree in respect to CHNF and with a rod-like appearance (Figure 1, Panel B, (a)), [57]. 

Nanochitin has received a crucial position in nanocomposite materials as the filler phase, due to its intrinsic properties [63]. The physiochemical and biological properties (light weight, small size, natural and biodegradable character, chemical stability, and non-cytotoxicity) of chitin at nanoscale dimensions make this material a valid candidate for utilization in food packaging and biomedical sectors [63], especially thanks to its high antibacterial effect [58,64,65]. Chitin is characterized by antimicrobial activity, this character is related to its chemical organization consisting of (1,4)-b-N-acetyl-D-glucosamine-replicating units. Likewise to starch and cellulose nano-fillers, a broad variety of nanocomposites exploited the interesting mechanical characteristics of nanochitin in polymeric-based systems [57,66,67,68,69].

Salaberria and co-authors [57] analyzed the effect of chitin nanocrystals (CHNC) and nanofibers (CHNF) at 5 and 20 wt % in thermoplastic starch matrices. The authors observed that the improvement of the final characteristics of the nanocomposites (superior barrier, thermal, mechanical, and antifungal characteristics) depended essentially on the morphological characteristics of the nanofillers used as the reinforcement phase. 

The chitin whiskers were found to enhance the water resistance and tensile strength of the neat polymer when assembled to soy protein [70]. Likewise, when chitosan whiskers were combined with chitosan films, it was noted that whiskers improved water resistance and tensile strength of the chitosan films [71]; however, when included in hydroxypropyl and carboxy methylcellulose, were capable of enhancing the mechanical and barrier functions of the films [72,73]. Research has also shown that nanosized chitin can improve barrier properties when embedded in a polymer matrix, such as starch or PVA [74,75]. 

#### 2.2.2. Chitosan Nanoparticles 

Nanochitosan is a green extract with exceptional physicochemical characteristics; it is characterized by bioactivity that does not damage humans [76]. It is largely utilized as a controlled release drug carrier, and, for gene transfer, Nanochitosan has been applied to realize systems with improved strength and wash ability of textiles, conferring antimicrobial effects [77]. Chitosan at the nanoscale can be realized considering precipitation or coagulation, ionic cross-linking, emulsion droplet coalescence, and covalent cross-linking procedure. Berthold and co-authors [78] obtained chitosan particles by considering sodium sulfate as a precipitation agent and incorporating a dispersant (Tween 80) to the chitosan acidic solution. Tian and Groves and co-authors [79] enhanced this procedure and obtained chitosan nanoparticles (CSNPs, 600–800 nm). Ohya and co-authors [80] proposed the use of glutaraldehyde to cross-link the free amino groups of chitosan; this was a water-in-oil (W=O) emulsifier, realizing 5-fluorouracil (5-FU) chitosan particles (size: 0.8–0.1 mm).

Kongkaoroptham and co-authors [37] proposed the variation of chitosan (CS) poly(ethylene glycol) methyl ether methacrylate (PEGMA) (Figure 1, Panel B, (b)), prepared by radiation-induced graft copolymerization, as a new compatible bio-based filler at the nanoscale level. The nanoparticle dimensions of PEGMA-graft-CSNPs were distributed from 30 to 100 nm. The strategy was studied to improve the compatibility, the mechanical, and the thermal characteristics of poly(lactic acid) (PLA). The mechanical properties of the PEGMA-graft-CSNP/PLA blends showed an improvement in terms of deformation at break, and a reduction of the tensile modulus brittle behavior to a more ductile behavior. In the case of poly(butylene adipate-co-terephthalate) (PBAT) films, antimicrobial packaging systems, combining different quantities of chitosan nanofibers (CS-NF), have been obtained by applying the solvent casting procedure; the biocomposites had high stiffness, strength, and glass transition temperatures, and low ductility, water vapor, and oxygen permeability. For all the nanocomposites, the migrated quantities in polar and non-polar food simulants were significantly under the overall limits recognized by the present legislation on food contact products. The produced films showed antimicrobial effect towards foodborne pathogens [81]. 

### 2.3. Nanofillers from Proteins

Proteins are another class of biomaterial largely investigated as a valid material to modulate functional properties in biomedical and packaging applications. Specifically, in this section, the authors focused their attention on the characteristics of keratin and gelatin. 

#### 2.3.1. Nanokeratin

Keratins are natural proteins [82] mostly diffused in poultry feather horns of animals, hair, and wool [83]. Keratin-based biomaterials have been essentially studied to realize hydrogels, films, scaffolds, and dressing, which were applied to get several biomedical disposals, as well as wound healing, cell culture, bone, and nerve regeneration, due to their essential biocompatibility and biodegradability [84,85]. Keratin obtained from human hair have been established in enhancing survivability in multiple animal models of bleeding and the efficacy in arresting hemorrhage [86,87]. Human hair keratin was utilized for the first time to realize hemostatic disposal, as documented in a Chinese medical book named Ming Yi Bie Lu in the 5th century [83]. In the last years, keratin products extracted from human hair have been used and applied to realize nanoparticles, hydrogel, sponge, and fibers, to develop hemostatic agents [88].

Fabra and co-authors [38] investigated the combination of nanokeratin obtained from poultry feathers, applying chemical treatment with polyhydroxyalkanoate (PHA)-based materials, following different strategies. Nanobiocomposites with high-barrier properties, based on the mixture of PHAs with nanokeratin, showed different morphologies, such as spherical nanoparticles and fibrillar sizes (Figure 1 Panel C, (a)). They were productively designed and realized via both: (i) direct melting technique; and (ii) pre-incorporated into an electrospun masterbatch of PHA, which was then melt compounded with PHA. Improved barrier characteristics for nanocomposites were experientially investigated and were seen to be related to PHA grade. Secondly, nanokeratin films, extracted using the solvent casting technique, were hydrophobized by coating them with electrospun PHA fibers. The multilayered selection was characterized by good adhesion and led to the improvement of the ultimate barrier properties.

#### 2.3.2. Nanogelatin 

Gelatin is a biodegradable protein extracted from natural sources by applying acid- or base-catalyzed hydrolysis of collagen. Gelatin is largely used in biomedical application. It is a polyampholyte macro-molecule characterized by the presence of anionic, cationic, and hydrophobic groups [89]. Gelatin molecules have repeating strings of alanine, amino acid triplets, proline, and glycine that influence the triple helical structure of gelatin. The high reliability of gelatin is due to its exceptional triplehelix organization, due to three polypeptide chains [90]. 

The properties of gelatin nanoparticles (GNPs) can be maximized by applying a particular extraction procedure. GNPs can be extracted by applying: (i) two-step desolvation, the method is characterized by the presence of a desolvating agent in an aqueous gelatin solution, resulting in conformational modification from triple helical coiled macromolecular arrangement to nanoparticles. Kumari and co-authors organized GNPs in a size range of 110–257 nm by this procedure [91]. (ii) Simple coacervation: Mohanty and co-authors [92] successfully obtained stable spherical nanoparticles (45 ± 5 nm) by measured presence of ethanol to aqueous gelatin solution. (iii) Solvent evaporation: this methodology is characterized by a single emulsion, oil-in-water (w/o), or double-emulsion, (water-in-oil)-in-water (w/o)/w, procedure. Water solutions that contain drug and gelatin are incorporated with ultrasonic treatment or high-speed homogenization with the oil phase. The water-in-water mixture method is utilized to produce insulin-loaded GNPs (250 nm) in mild conditions for the activity of insulin [93]. (iv) Microemulsion: It is a new and successful procedure used to prepare gelatin particles. In this process, gelatin in aqueous solution is combined to the solution of surfactant [sodium bis (2-ethylhexyl) sulfosuccinate (AOT)] in n-hexane and then glutaraldehyde (GA) to cross-link the nanostructures, followed by evaporation of n-hexane for recovery of GNPs [94]. The nanostructures had dimensions ranging 5–50 nm. (v) Nanoprecipitation: Implies the precipitation of pre-formed GNPs from an organic solution and the dispersion of the organic solvent in the water solution with prevalence of a surfactant [95,96,97].

The diverse aspects of gelatin nanostructures influence the particle properties, like polydispersity index, zeta potential, drug release, and entrapment efficacy characteristics [98]. In the field of packaging [99], combination with chitosan has been found: Kumar et al. [100] efficiently made hybrid nanocomposite films realized with chitosan, polyethylene glycol, gelatin, and silver nanoparticles (AgNPs), applying a solvent casting procedure, and the researches on packaging of red grapes underlined that the shelf life of the fruit was prolonged for a supplementary two weeks in case of the hybrid film. Other than blending, gelatin as nanoparticles can find application in the food sector as nanocarrier systems for the controlled delivery of a variety of food supplements and additives.

## 3. Conventional Matrices for Packaging 

**Figure 2:**
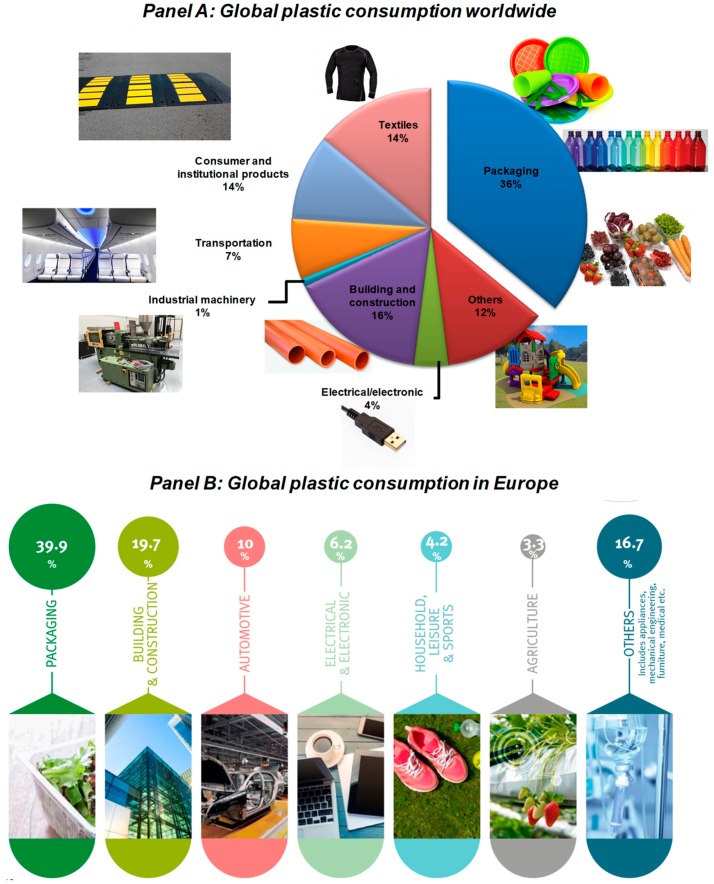
Panel **A**: Global worldwide plastic production and related application sectors; Panel **B**: Global plastic consumption in Europe [101].

The conventional matrices are used in different sectors: packaging, textiles, construction, electronic, transportation, etc. Figure 2 shows the global plastic production worldwide (Panel A) and in Europe (Panel B) in relation to the different application sectors. Packaging is one of the main important sectors that supports the use and application of polymeric materials derived from fossil sources, with an annual global demand at around 36% [1], followed by the building and construction sector, with an annual request of 16%, while the total consumption of plastic in Europe is at around 39.9% [101]. The packaging sector is influenced by the demands requested by various stakeholders (consumers, producers, and retailers), who have specific necessities and do not, each time, recognize the packaging as an additional significance to the product [102]. The conventional characteristics of packaging are centered on the protection of food products from degradation processes (mainly induced by several factors such as temperature, light, moisture, and oxygen conditions of the environment), to enclose the food, and to supply consumers with ingredient and nutritional information and the description of several items of information regarding the conservation of food products [103,104]. In addition, the packaging system needs to improve the shelf life of packaged food, preventing the deterioration and the organoleptic/external (color and esthetic characteristics) qualities of products [19,23]. These concepts have constantly been related with an inert substrate, acting as a barrier substrate between the food and the outside atmosphere, reducing and eliminating the passage of dangerous substances from the packaging to the food [20,24]. 

Conventional matrices have long been crucial materials in packaging and in food packaging, due to numerous reasons, with their easy processability, low cost and essentially for their mechanical performances. In the first half of the 20th century, thermally processable polymeric matrices were developed, studied, and applied to packaging to restore glass, paper, and metals (foils and laminates, aluminum, tin-free steel, and tinplate) [104].

The petroleum-based matrices were developed and studied from the first half of the XX century. As an example, polyethylene (PE) was studied/synthesized in the 1930s and polypropylene (PP) in the 1950s, while polyethylene terephthalate (PET) and linear low-density polyethylene (LLDPE) were studied in the 1970s [105]. The common and most used food-packaging polymers are polyethylene terephthalate (PET) (applied in food, beverage, and other liquid containers), polyethylene (PE) (cooking oil, milk, and water containers), polystyrene (PS) (mushroom and eggs), polypropylene (PP), polyvinylchloride (PVC) (spice ice tea, yogurt and margarine), and polyamide (PA) (stretchy packaging of fresh food, such as cheese and meat) [102]. 

Table 1 summarizes the main important physical characteristics—glass transition temperature (Tg); melting temperature (T_m_); tensile strength (T); deformation at break (ε_b_, (%)); optical properties (OP), as well as transmission of visible light, haze, gloss, and permeability (water vapor (H_2_O), Oxygen gas (O_2_), and carbon dioxide (CO_2_))—of petroleum based polymers applied in packaging and the food packaging sector, highlighting at the same time the main applications. 

Polyethylene terephthalate (PET) is fundamentally applied in sectors requiring low permeability to gases, high mechanical performances (deformation at break and strength), wide temperature resistance (resistance at freezing temperatures, high softening point), and adequate transparency. High density polyethylene (HDPE) is an interesting polymeric matrix that represents more than over half of the food packaging available on the market. HDPE is mainly used in contact with a variety of foods, in addition it is easily shaped; low density polyethylene (LDPE) is extensively used in film to cover foods. High resistance to tear, low heat seal temperature, and low permeability to water (its deformation at break exceeds that of other commercial and largely used plastics) are the main required properties for the specific application. 

Polyvinyl chloride (PVC) shows good gas barrier performances and high chemical hardiness, this polymer is often considered as a valid option to cover meat and fatty foods. PP is largely utilized for containers and walled cups. Satisfied barrier properties, optical transparency and esthetic quality, strength, and high temperature resistance allow this polymeric matrix to be suitable for the realization of microwave/freeze containers, and sterilized or hot-filled containers [106,107]. Polypropylene (PP) is gradually substituting polystyrene (PS) in rigid thermally-processed cups (e.g., yoghurt) [105]. Nevertheless, the low barrier properties of PP makes it acceptable for packing limited shelf-life food (e.g., cheese); the high thermal properties suggested the possibility to realize PP in thermal insulation applications by using foams (e.g., disposable packages) and reduced weight, but still not flexible and rigid dishes.

Polyamides (PA) are engineered semicrystalline thermoplastics extensively utilized in the food packaging segment, thanks to good barrier characteristics to gas permeation, interesting mechanical and chemical resistance, good printability, and interesting optical properties in terms of transparency. The performances of the final plastic objects are, certainly, strongly related to the chemical structure of the PA, and also related to the selected process utilized to realize the products [108]. The aid of a comonomer can actually influence both intramolecular and intermolecular interactions, incorporating relevant modifications in the crystallization phenomenon of a PA.

Poly(vinyl alcohol-co-ethylene) (EVOH) is a semicrystalline random copolymer with elevated transparency, optical characteristics, and outstanding gas barrier performances to hydrocarbons [109], particularly when low content of ethylene (below 38 mol % ethylene) is considered [110], and excellent chemical resistance [111]. EVOH-based formulations have been progressively applied in food packaging, being the sector characterized by severe value/standard in terms of gas and chemical resistance, water hydrocarbon permeation, and aroma migration [112]. The physical (barrier and mechanical) performances of EVOH under dry environments are attributed to the elevated intra- and inter-molecular cohesive energy and semi-crystalline microstructure [113]. The disadvantage of EVOH copolymers is their moisture sensitivity, which influences negatively their characteristics (barrier, thermal, and mechanical properties) at high relative humidity [114].

To limit this disadvantage, EVOH in the food packaging sector is often utilized in multilayer polymeric systems, and combined with hydrophobic polymeric matrices, to improve the final characteristics of packaging systems [110]. 

PVA is one of the most popular synthetic polymers for packaging sectors, thanks to its good compatibility, processability, and acceptable thermal properties. It also possesses good chemical resistance and high mechanical properties, although its disadvantages include limited barrier and thermal properties and relatively high cost [115]. The properties of PVA generally depend on its molecular weight and degree of hydrolysis: many hydroxyl groups on the PVA surface makes it one of the most hydrophilic polymers with high moisture sensitivity, and hence its resulting blends and composite materials have become popular for packaging applications [116,117]. In general, full-hydrolysis PVA is not considered to be a thermoplastic polymer, mainly due to its melting temperature being very close to the degradation temperature in the absence of plasticizers. Therefore, it is essential to use plasticizers for PVA in order to control the relevant melting temperature, fluidity, and thermal stability, especially for screw extrusion and injection molding processes widely used for the packaging sector.

The triumph of polymeric-based systems in the packaging sector is related to the incessant progress of better performing neat polymers and polymeric based blends, with relevant progresses in polymeric matrix processes. In fact, a clever process control of the thickness of different systems permits the production of lighter packages, reducing the material cost [105]. The lamination and the coextrusion processes represent a valid opportunity to design and realize multilayer-based systems, selecting individual layers to ensure specific properties (mechanical resistance, barrier, and aesthetical); however, biaxial orientation increases, considerably, the mechanical and barrier characteristics of a film or bottle, positively influencing some attractive behaviors.

The process technology adopted using polymers are usually more energy-friendly in respect to the use of other materials (the working process temperature profile is above 300 °C, while the temperature profile used to process the plastic is typically below 300 °C). The packaging materials incorporate, sometimes, plasticizers, stabilizers, and fillers. Plasticizers are used to modulate the mechanical performance (ductility, strengthen, flexibility, and toughness of polymers; though induced a reduction of stiffness and hardness) [43,123], while stabilizers are included into the polymers to moderate the reduction of mechanical characteristics induced by UV light and oxygenation, and fillers tend to improve or preserve the mechanical and barrier performances in respect to the neat/control [24,124]. In addition, technological developments have permitted the reduction of the weight of packages (at around 28% in the last few years [105]), which in turn induces relevant savings not only in terms of transportation costs, but also in terms of environmental issues. Regarding this issue, in the last decades, the growing environmental contamination brought by the high impact of plastic wastes based on petroleum extracts has attracted the attention of industrial and academic researchers to design and develop some innovative polymeric systems. In this context, bio-based polymers have attracted some interest in respect to conventional ones. Green polymers symbolize a strategic option to design and realize new eco-friendly and sustainable systems that are able to reduce/minimize the plastic wastes stored every day in landfills, and the emission of greenhouse gases (GHG), which strongly depend on fossil extraction, production, use, and end-life [24,125]. There is an increasing interest worldwide to substitute traditional plastics with bio-based ones, mainly in packaging sector. The utilization of bio-based materials and resources is seen as one of the numerous strategies able to reduce the environmental impacts induced by the use of petroleum-based extracts. 

Future prospective in the polymeric package sector will be influenced by: (i) How these resources will guarantee the increasingly additional rigorous necessities for packages; (ii) the processability of novel bio-based/biodegradable polymers; and (iii) legal, market, and environmental issues.

## 4. Bio-Based Matrices for Packaging Applications

Presently, packaging films are typically constituted by petroleum-based synthetic polymeric matrices that monitor the market, due to their reduced price and simple accessibility. As already analyzed, these polymeric matrices comprise polyolefins (polyethylene), ethylene vinyl alcohol (EVOH), that are responsible for a relevant barrier towards water and oxygen. Nevertheless, they are hindered by margins in petroleum resources and the lack of bio-disintegration, which magnify the ecological and cost-effective concerns. 

**Figure 3:**
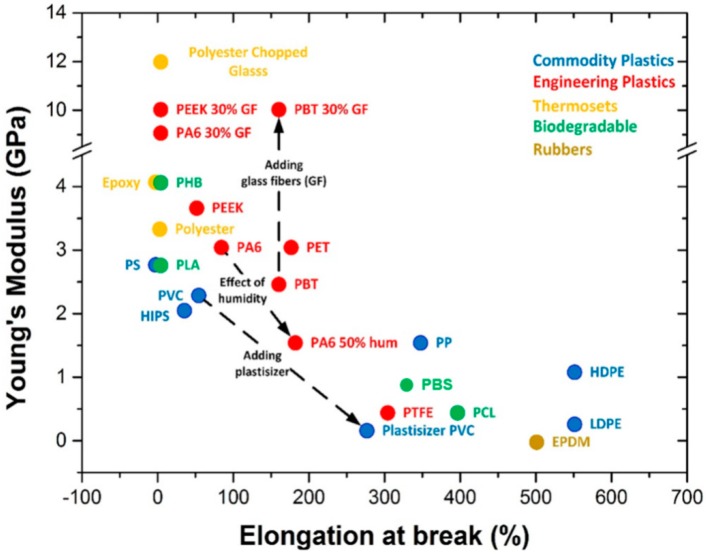
A comparison of the intrinsic properties (mechanical characteristics) of usually utilized plastic materials, engineered polymers, rubbers, bio-based polymers, thermosets, and plastic materials [127].

Bio-based polymers are considered a valid replacement of petroleum synthetic polymeric matrices; they are obtained by the processing of renewable resources (vegetable and animal wastes) and offer several positive aspects, such as environmental advantages, disintegrability and degradability, improved possibility to recycle the polymeric wastes, no presence of toxic components, and high biocompatibility, in respect to the conventional petrochemical polymeric matrices. The interesting barrier properties of different bio-based polymeric films for package designs has been acknowledged in several research review articles [126,127]. Due to the fact that numerous biopolymers show water affinity, their barrier and mechanical characteristics are subjected to the humidity and ambient atmosphere, which may decrease their overall performance and the quality of packages when compared with petroleum-based polymers (Figure 3). 

Furthermore, molecular weight, physical properties (crystallization phenomenon and crystallization degree), visco-elasticity, and rheological characteristics may induce disadvantages, thus several modifications or adjustments during the processing steps are necessary to modulate the final performances. Consequently, biopolymers should be modified, studying new polymeric blends combining two or more different polymeric matrices or fillers, at the micro/nanoscale level, to expand their characteristics/properties, especially dealing with nature and processability behavior. 

Bioplastics can be classified into three important classes, in function of their source: non-biodegradable bio-based bioplastics (e.g., polyethylene terephthalate (PET), PA); biodegradable bio-based bioplastics (e.g., PLA, polyhydroxyalkanoates (PHA) or starch, other polysaccharides or proteins); or fossil-based biodegradable plastics (e.g., polycaprolactone (PCL)) [128] (Table 2). 

**Figure 4:**
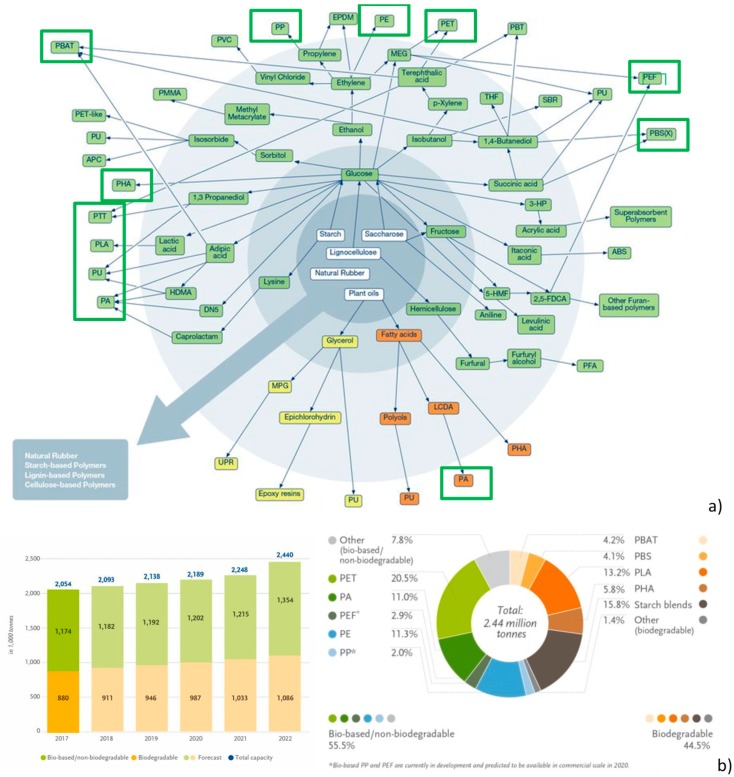
Commercially realized pathways from biomass via different building blocks and monomers to bio-based polymers (**a**); estimated global production capacities of bioplastics (biodegradable, bio-based/non-biodegradable) for 2022 by material type (**b**) [131].

Nevertheless, the general principle to be considered to classify biodegradable materials is the raw material origin and their production step. In accordance to this, biodegradable polymers are categorized into three groups: “1^st^ class” or biomass derived polymers, including cellulose acetate, cellulose, chitin, and starch; “2^nd^ class” or biopolymers synthesized utilizing microorganisms and plants, as poly(hydroxy alkanoates (PHAs); and “3^rd^ class” or synthetic polymeric matrices, as polylactide (PLA), poly(butylene succinate) (PBS), bio-polyolefins, bio-poly(ethylene terephtalic acid) (bio-PET), and synthetic polymeric matrices chemically produced from renewable sources [130] (Figure 4a,b).

Typically, 1^st^ class is utilized avoiding the refining step, whereas 2^nd^ class polymeric matrices are obtained from natural extracts, and they play a central position in conditions that necessitate biodegradability. The utilization of 1^st^ and 2^nd^ class polymeric matrices permits a more proficient manufacture, with materials having preferred and modified physical and functional characteristics, but limited flexibility in the chemical structure. Monomers utilized in 3^rd^ class polymers are obtained by modifying natural molecules or by the chemical modification of natural macromolecules combining and applying chemical and biochemical technologies. Several of these 3^rd^ class polymeric matrices, such as bio-PET and bio-polyolefines, will not go into natural cycles after utilization, so their concurrence to the reduction of environmental impact is mainly related to the decrease of the carbon footprint. Studies and researches regarding progress, innovation, and applications of the three classes of polymers are described in the following sections, their main important characteristics and chemistries are also described. 

A selection of polysaccharides and their modified products have been utilized to realize biodegradable films to be used in packaging and edible coatings. These carbohydrates composed by glycosidic bonds are one of the most important structural components of vegetables (e.g., cellulose) and animal exoskeletons (e.g., chitin), and they can have, in addition, a crucial function in the “green” energy storage (e.g., starch) [132]. With growing consideration and research on this area, it has been practicable to open the way to overtake their intrinsic limitations and identify solutions [133]. In the following paragraph, some of the principle utilized polysaccharides in food packages are summarized.

### 4.1. Starch 

It is composed of amylose and amylopectin polymers (α-d-glucose monomers), containing hydroxyl groups (–OH). These groups show strong intermolecular interactions, which give, on the basis of tighter arrangement, increased crystallinity and melting temperatures. As a consequence, starch molecules are thermally affected, which strongly limits their application as a packaging material (Table 3) [134,135]. Alternatively, the presence of hydroxyl groups supports the break of hydrogen bonds that induce the disintegration into small fragments. Starch products are food sources for microorganisms in determined environment conditions, as elevated humidity (~55–60% RH), in the presence of satisfactory oxygen feedstock, and suitable temperatures to make the biodegradation easy [136,137]. In addition, starch hydrophilicity creates materials with a poor water barrier [138]. At a modest or elevated relative humidity, starch-based substances tend to soak up moisture from the environment. Firstly, this behavior will induce swelling of the matrix, with disruption of hydrogen bonds, after that the increase of free volume spaces will also enhance the chain mobility. Therefore, moisture and gas barriers are negatively influenced and, consequently, are drastically compromised. According to this, starch may not be adequate to realize packages for dry and oxygen-sensitive foods. Other than low barrier properties, starch materials have the inclination to show reduced mechanical characteristics, reducing, consequently, again their utilization [139]. Furthermore, even if starch price is relatively low, adaptation of its performances may improve the final cost of different materials. Numerous parameters should be considered for the design of starch food packages. 

Regardless of several published research articles that developed and studied TPS (thermoplastic starch) products, the weaknesses, such as reduced barrier and mechanical performances, are still unsolved. As a consequence, additional strategies, such as the compatibilization, chemical and physical modifications, and development of polymeric blends, have been suggested to solve the disadvantages of using TPS. Polymeric blends combining two or more polymers are a valid possibility to modulate and improve TPS performances. This approach is considered cost-effective, since no modification is required and a wide frame of polymeric material processability can be opened.

### 4.2. Cellulose 

Cellulose (Table 3) shows regular arrangement and structure of hydroxyl groups, it is characterized by the tendency to organize crystalline microfibrils with strong hydrogen bonds. It has specific interesting characteristics, such as high mechanical strength, low density, high durability, low price, no presence of toxic elements or substances, biodegradability, interesting and easy chemical modification, and stability [141]. In the textile sector, the application of cellulose is largely utilized in packaging and fibers, and can be classified into two groups: modified and regenerated cellulose [142]. Different chemical modifications, such as etherification and esterification, are frequently and typically considered to improve the thermal processability cellulosic materials. Numerous modified celluloses are commercially available, the main ones are cellulose esters (for melt compounding), cellulose acetate, and regenerated cellulose for fibers [143]. The application of plasticizers and polymeric blends are also evaluated considering that the chemical and mechanical characteristics are largely influenced by the blend composition and technical processability followed and applied for their realization [144]. 

### 4.3. Chitin/Chitosan 

Chitin (Table 3) is a natural polymeric material that composes the exoskeleton of arthropods, it is also present in the cell walls of yeasts and fungi. In addition, it is an acetylated polysaccharide made up of N-acetyl-d-glucosamine. Chitin is present also on the market after a chemical treatment (extraction from crabs and prawns wastes). Chitosan is indeed extracted after the treatment of chitin, applying a deacetylation process in which several parameters that should affect the extraction and also modify the main characteristics (e.g., temperature, native origin of chitin, and alkali concentration) need to be controlled. Chitosan does not show affinity to water, in fact it is insoluble in water, but may be without problems dissolved in acidic aqueous solutions. The interesting film-forming characteristics permit the realization of coatings and membranes capable to be utilized for food conservation [145,146]. 

The membrane of chitosan is characterized by modest water permeation and low oxygen permeability, crucial in the conservation and preservation of some food that are sensitive to the presence of oxygen in the packaging [147,148]. 

The development of polymeric blends composed by chitosan and other polymeric materials, such as starch [149] and proteins [150], represents a valid approach to guarantee an enhancement in terms of mechanical performances, improved characteristics in terms of lower water solubility, and water vapor permeability. Several other approaches, for instance coating, dipping, casting, Layer-by-Layer (LbL) assembly, and extrusion, have been considered to realize chitosan systems with several characteristics. The promising food sector of chitosan systems as antibacterial active compounds and sensing and barrier systems have had enormous progresses [151]. 

### 4.4. Biopolyesters from Microorganisms—Polyhydroxyalcanoates (PHAs) 

**Figure 5:**
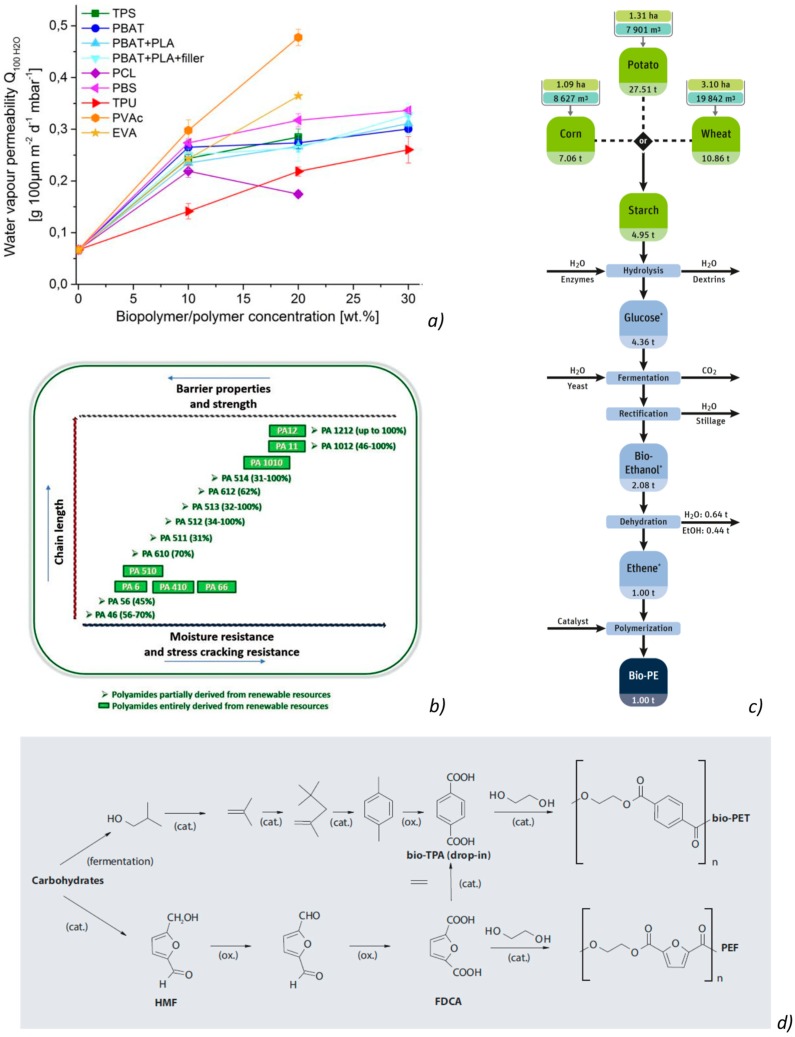
Water vapor permeability for Poly(3-hydroxybutyric acid-co-3-hydroxyvaleric acid) (PHBV) blended with nine different biopolymers and polymers at different concentrations (**a**) [168]; polyamides completely and partially obtained from renewable sources (**b**) [163]; processing route for bio-polyethylene (bio-PE) (**c**) [169]; (**d**) transformation of sugar to Polyethylene 2,5-furandicarboxylate (PEF) and bio- polyethylene terephthalate (bio-PET), a new bio-based plastic similar to PET [170].

PHAs are bio-based polyesters produced by microorganisms as a carbon source under nutrient stress conditions. PHAs are realized after the fermentation process of different materials (sugars, organic, agricultural, and municipal solid waste, etc.) [152]. This class of biopolyesters is biocompatible, biodegradable, and thermally processable. PHAs are utilized in food packaging application to realize coatings, films, boxes, foam materials, and fibers [153]. The final characteristics of the PHAs are influenced by monomer composition, carbon nature, and source and kind of microorganism utilized during the fermentation. Within PHAs, polyhydroxybutyrate (PHB) homopolymer has a high degree of crystallinity, resulting in a stiff and brittle nature. PHAs are resistant to hydrolytic degradation, PHAs show interesting and exceptional film-forming ability and offer a low permeation to gases (oxygen and water vapor), good UV resistance, but low chemical resistance towards acids and bases [154]. Two main restrictions for their use and application in large-scale are related to the high cost of polymers and characteristics (they suffer problems during processing because of the limited temperature processing range and a moderately low-impact performance related to high crystallinity values). Some disadvantages of PHB for industrial applications may be overtaken by copolymerization with hydroxyvalerate or hydroxyhexanoate. Monomers can be used in 150 different combinations to produce copolymers with diverse properties. PHB polymeric matrices showed some different characteristics, modulating the copolymers ratio. PHB can be less crystalline and more elastic in the presence of long alkyl side chains, such as hydroxyvalerate (HV) and hydroxybutyrate (HB), that provide, respectively, P(3HB-co-3HV) and P(3HB-co-4HB) [125]. The polymeric blends based on PHB, combined with other polymeric matrices, can influence and encourage its use (Figure 5a) [153]. For example, PHB has been combined with poly(vinyl butyral), poly(vinyl acetate), poly(ethylene oxide), cellulose acetate butyrate, poly(vinyl phenol), chitosan, and chitin. Distinctive characteristics of this set of polymeric matrices are outstanding rigidity, heat, and chemical performances. Isotactic polypropylene (PP) showed characteristics comparable to the cited PHB copolymers. Isotactic PP showed remarkable water vapor resistance, in respect to the bio-based polymeric matrices present in the market. For all these characteristics, bio-based polymeric matrices may be used in several applications, including packaging.

### 4.5. Biopolyesters from Biotechnology and Conventional Synthesis from Synthetic Monomers

#### 4.5.1. PLA 

(Poly lactic) polymers are obtained by the fermentation process applied to agricultural wastes and byproducts, such as starch-rich materials (wheat, corn starch, and maize). The procedure implied the transformation of corn natural resources into dextrose and following fermentation into lactic acid. PLA is a biodegradable aliphatic polyester and thermally-processable matrix, having interesting properties for the packaging sector [155]. The monomers based on lactic acid are also polycondensed or synthetized by ring-opening polymerization of lactide [156], and the properties are strongly related to the content of optical isomers of the lactic acid. High crystalline polymers are composed of 100% L-PLA monomers, while the different concentration of 90/10 D/L copolymers is considered to facilitate the processing above the glass transition. PLA is the first biodegradable polymer present on the market and largely commercialized. (Poly lactic) can be utilized to realize injection-molded coatings, films, and objects. In this context, PLA has substituted polyethylene terephthalate (PET), low-density polyethylene (LDPE), high-density polyethylene (HDPE), and polystyrene (PS) in the packaging sector. PLA is several times utilized to realize single use material: blister packages, cold drink cups, lids, containers, thermoformed trays, bottles, as well as flexible films [157]. Even though PLA is, today, advantageous from an economic point of view, and it also has many favorable characteristics for packaging uses (i.e., simple processability, high transparency), it also shows some disadvantages, such as mechanical characteristics and poor barrier properties, which inhibit its industrial utilization [158]. Significant academic and industrial energies have been dedicated to the improvement of PLA characteristics to enlarge the application of PLA in different commercial applications of crucial importance in the industrial sector, in order to develop ecofriendly disposable materials. In addition, modulation of PLA properties by combining PLA with different polymeric matrices, is realized by melt blending, being this procedure is quite simple. Furthermore, the technologies required for the process are available at industrial level with limited costs. The technique permits the realization of simple packages with modulated characteristics by changing the content of different polymeric matrices, which are utilized to develop the blends.

#### 4.5.2. PBS 

Conventionally, poly(butylene succinate) (PBS) is obtained from succinic acid and 1,4-butanediol through catalytic hydrogenation of maleic anhydride. Succinic acid can also be synthesized by microbial fermentation. PBS can be degraded via ester linkages, and retains exceptional mechanical characteristics that permit it to be prepared by using conventional melt processes. Its uses consist of bags, hygiene commodities, and mulching. Their mechanical performance is inferior to polymeric matrices extracted from petroleum. Consequently, aliphatic polyesters could be utilized as transparent thin systems for packaging bags, filaments, blown bottles, agriculture, and thermo-processed or injection-molded systems. In order to enhance their properties, new polymeric blends or chemical modified copolymeric systems (aliphatic-aromatic copolyesters: poly(butylene terephthalate-co-succinate) (PBTS) and polybutylene adipate terephthalate (PBAT) can be realized. Copolymers based on polyesters are frequently composed by terephthalic acid to gain better processability and mechanical performance. Aliphatic-aromatic blends and copolyesters merge the fine useful characteristics of aromatic polyesters and the interesting biodegradability of aliphatic polyesters. Furthermore, the combination of characteristics permits the modulation of the performance of the final product, and address them to the utilization in the industry dedicated to the production of packages. The aliphatic or aromatic polyesters are based on petrochemical extracts and are usually prepared through conventional polycondensation methods. 

#### 4.5.3. PBAT 

Polybutylene adipate terephthalate (PBAT) is composed of a linear polymeric chain made by two comonomers (forming a copolyester): a rigid unit consisting of terephthalic acid and 1,4-butanediol monomers, and the elastic unit composed by adipic acid and 1,4-butanediol monomers. PBAT shows interesting thermal and mechanical characteristics when the amount of terephthalic acid is more than 35 mol %; nevertheless, the increasing amount is followed by an important reduction, considering the rate and the percentage of biodegraded quantities [159]. This aspect is related to the existence of aromatic polymeric chains, which build these materials up to be more resistant against microbial attacks [160]. PBAT is also extremely flexible and soft, to make it applicable in films (mulch), blown bottles, and injection-molded and thermoformed systems and filaments. It is extensively utilized for disposals characterized by a short life, such as food films and compostable systems. This copolyester is commercially utilized for the realization of single-use not reusable packages, organic waste bin liners, compostable bags, and protective plastic systems [161].

### 4.6. Bio-Polyamides 

Castor oil is the raw material used as a renewable feedstock for the production of commercially available bio-based polyamides. Castor beans are characterized by an unusually high amount of ricinoleic acid (40–60%). The presence of hydroxy groups and double bonds of the acid determine numerous opportunities for its chemical modification. Chemical modifications permit the synthesys of different blocks, such as aminoundecane (decamethylenediamine) and sebacic acid [162]. As in the case of standard polyamides, bio-PAs are frequently produced by polycondensation of dicarboxylic acids with diamines, by ring-opening polymerization of lactams, or polycondensation of amino acids [163]. Monomers are mostly obtained from fossil oil, but can even be from biomass (Figure 5b). There is an available patented method that considers PA6 production by ring-opening polymerization of ε-caprolactam, obtained by glucose fermentation, from totally renewable feedstocks, such as sugar [163]. The same bio-based methodology can be utilized to obtain PA 66. In this case, adipic acid, extracted during the fermentation process of glucose or plant-oil and hexamethylenediamine (HMD), which can be quickly extracted from biomass, can be used.

### 4.7. Bio-Polyolefins 

Bio-based polypropylene can be produced by using bio-ethanol (Figure 5c); however, this process is relatively more complex and involves several ways of obtaining the propylene monomer C_3_H_6_ from various renewable resources [164,165]. Major applications of bio-based polyolefins include packaging, building and construction, automotive and transportation, and others including blow molded bottles, stretch and shrink films, and detergents. Packaging emerged as the leading application segment on account of shifting demand from synthetic polymers to bio-based polyolefins in 2013. Automotive and transportation is the second largest market for bio-based polyolefins, owing to its increased application in manufacturing automotive parts. Moreover, building and construction is expected to witness strong growth in the bio-based polyolefins market.

### 4.8. Bio-Poly (Ethylene Terephtalic Acid) (Bio-PET or PEF) 

The furanic–aliphatic polymeric class bas been largely investigated in recent years. These polymers based on ester groups are characterized by the presence, in their backbone, of aliphatic and furan units (i.e., obtained from an aliphatic monomer and FDCA (2,5-furandicarboxylic acid)) (Figure 5d). They have been custom-made by means of several aliphatic monomers, counting those with a linear carbon sequence or those with an extra rigid cyclic arrangement [166]. Poly(ethylene 2,5-furandicarboxylate) (PEF) is currently considered as an attractive sustainable substitute of poly(ethylene terephthalate) (PET), due to the fact that it has better barrier and interesting thermal characteristics (e.g., lower melting phenomenon and higher glass transition temperature) than PET. The reduced permeability of PEF to CO_2_, O_2_, and H_2_O is a great benefit for packages. Ii is expected to enter the market in 2020 to replace PET [167]. 

## 5. Hybrid Blends Based on Bio-Based and Fossil Fuel-Derived Polymers

Natural-based polymeric matrices are largely susceptible to moisture and, according to this, do not afford a good barrier to gas diffusion. Hybrid blends containing renewable polymers in combination with man-made polymers and additives have shown great potential in solving some of these limitations. In fact, most of the commercial food packaging is based on hybrid materials, giving the required properties and functionality to a variety of foods [171].

Bio-based plastics are characterized by costs much higher than traditional thermally-processable polymers (e.g., PP and HDPE), consequently, it is not advantageous to use them without combining these two products. In general, different parameters influence the final microstructure characteristics and mechanical performances of not totally miscible polymeric blends, thus it is evident that the overall performance of polymeric blends is strongly connected to blend composition and its phase morphology [172].

One of the most important aspects of such kind of blending is the recyclability, due to the fact that bio-based plastics could, on one hand, interfere with the current recycling of plastics, and hence hinder the closure of plastic cycles [173] (end-of-life compostable bioplastics may pollute recycled plastic streams if not properly separated and managed, which is undesirable given the current attention on a transition towards a circular economy), while on the other could enhance the recalcitrance of fossil-based mixtures to disintegration and compostability [174,175,176]. Nevertheless, even if the mechanical recycling of bio-based non-biodegradable plastics, such as bio-PP, bio-PE, and bio-PET, is chemically matched to their fossil counterparts, thus totally compatible with the current recycling methodology, a limiting issue is that these materials (as well as blends of bio-based and fossil plastics) are not compatible with sorting in single polymer streams, even in larger quantities. Therefore, if not mechanically recycled, they can only be incinerated with energy recovery (or anaerobic digested with biogas production).

Materials obtained by mixing synthetic polymers with natural polymers can potentially compromise environmental, economic, and social aspects by reducing the recyclability when compared to traditional polymers [177,178]. Therefore, a main concern associated with the strategy of introducing natural/biodegradable polymers into conventional polymers is related to the chance that these blends have lower levels of recyclability compared to the original polymers, which would then have an adverse impact on both environmental and industrial issues.

It should be also recognized that use of bio-based or biodegradable plastics is often justified by asserting that they biodegrade faster than their conventional petrochemical counterparts; on the other hand, it has to be considered that new demand for biomass inputs could negatively expand uses of land, fossil fuels, chemical inputs, and water. Additionaly, bioconversion may require the use of potentially toxic petroleum-based solvents and results from life cycle assessments could give high energy consumption and emissions for bio-based polymers. When deciding to fully or partially replace conventional petroleum-based plastics with bio-based plastics, it is important to understand the flow of these materials and their adverse impacts in all parts of their life cycles in order to select a material that is more sustainable [179].

Given, as recognized, that the characteristics of biopolymers must be improved substantially if we want a penetration in the market, the change of these materials toke the attention of researches. In a dissimilar manner to the design of new polymers and polymerization routes, polymer blending is a comparatively low-priced and fast process to modify the characteristics of polymeric materials. As a consequence, this procedure may take part in a critical manner in improving the attractiveness of bio-based polymeric matrices [180]. Further studies are requested to optimize the miscibility of these systems to exploit their potentials. It is widely reported in the literature that different chemical modification routes, such as grafting, transesterification, and copolymerization, have been developed to obtain polymeric materials and blends with valuable characteristics. 

### 5.1. Starch

In the case of starch-based blends [181], numerous studies have reported, even recently [182], the blending of thermoplastic starch with other man-made plastics (e.g., polycaprolactone, polyamide, polyolefins) [183]. While the blending approach reduces some limits of the TPS systems, the successful approach in improving adhesion can be found by considering non-reactive mixing (blending TPS with graft block or random copolymers) or reactive compatibilization (by polymerization, grafting, or branching). 

**Figure 6:**
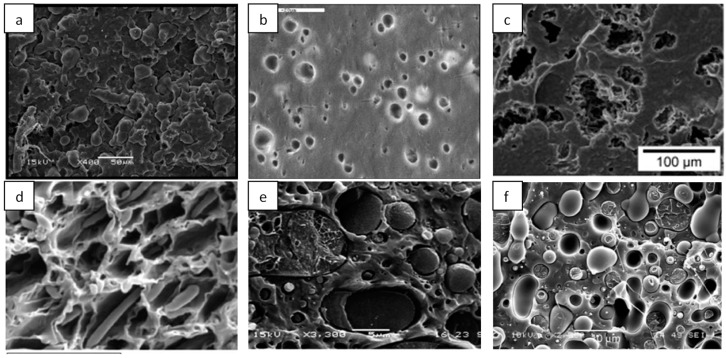
SEM micrographs of (**a**) PP/TPS/MA blend [184]; (**b**) TPS/PE compatibilized with PP-g-MA 20%—scale 20 μm [186]; (**c**) 30TPS/70PA12 blend [190]; (**d**) LDPE/PHB 80:20—scale 40 μm [191]; (**e**) PS45/PHB45-(block copolymer) C10 blend [192]; (**f**) PLA/PS (0.3 volume fraction) blend [193].

As an example, a comparison between the effects of carboxylic acids (stearic, palmitic, and myristic acids) and maleic anhydryde (MA)-grafted poly(propylene), used as a compatibilizer agent for PP/TPS polymeric blends, was conducted: Carboxylic acids revealed comparable compatibilization performance with respect to MA-grafted poly(propylene), regarding improved adhesion and better mechanical properties (Figure 6a) [184]. It was studied that carboxylic acids induced enhanced crystallinity in PP/TPCS blends. Polymeric blends based on the combination of traditional polymeric matrices and starch can also speed up the disintegration of traditional based systems used to realize packages [185,186]: The presence of different amounts of starch into traditional polymeric matrices can decrease the price and enhance the degradation of the blends, due to the fact that, if polymeric blends are buried in soil, the starch phase is degraded by microorganism attacks. This enhanced degradability due to the presence of porosity and voids induces the loss of integrity of polymeric systems (Figure 6b). Nguyen et al. [187] analyzed the behavior of LLDPE/TPS systems, obtaining a degree of disintegration of 63%. The systems disintegrated into methane, H_2_O, CO_2_, and biomass after five months in a composting environment. The results confirmed that LLDPE/TPS blends were disintegrated more rapidly than pure LLDPE, an enhancement in terms of porosity structure with a loss of integrity of the polymeric matrices was evidenced, finally achieving broken plastic small pieces. Consequently, if the PE part of the material is not modified to facilitate its disintegration and the subsequent biodegradation, only the starch part of the blend will biodegrade with fragmentation of the material as a result. However, it is highly questionable and hard to accept, as polyolefins present in the blends do not undergo biodegradation and are still present in the form of a polymer material when the biodegradable part of the blend degrades to water, CO_2_, and biomass. In the environment, the packaging product made of this kind of blend is only fragmented to small fractions of plastic that can be definitely dangerous for living organisms [188]. The recycling opportunity still represent a possible solution to this problem, as it has already been demonstrated that the replacement of products based on LDPE with LDPE/TPS blends may not result in substantial changes in the recyclability issues associated with the reprocessing of the involved systems [189]. 

In the case of PE, further investigation is mandatory to utilize LDPE-starch blends as bio-based packages, because of the decrease of the mechanical characteristics of PE when the starch amount increases [194]. Generally, the mechanical performance is reduced with the presence of starch because of the limited compatibility between PE and starch. The reduced compatibility is strongly related to the fact that starch granules are extremely hydrophilic, due to the presence of hydroxyl groups at their surfaces, while LDPE generally does not show affinity to water. In several researches, if the content of starch is increased at around 20 wt % the deformability of the PE matrix is changed and converted into a fragile material (in PE/starch based formulations) [195]; even gas diffusion and the water vapor transmission rate (WVTR) is changed in relation with the starch ratio, according to Arvanitoyanis et al. [196], that found how WVTR is decreased proportionally when the amylose content increases, due to amylopectin degradation during the thermal processing.

Pedroso and coworkers [197] and Rosa et al. [183] observed immiscibility between TPS and LDPE, Euaphantasate et al. [198] produced LLDPE/TPS systems by extrusion, presenting different amounts of starch, from 10 to 40 wt %; the microstructure shows the presence of two polymeric phases and micro-voids. 

Rare papers can be found for the realization of TPS blended with high melting synthetic polymers. In the case of polyamides, Landreau et al. [199] and Teyssandier et al. [190] investigated TPS-based systems combined with polyamide 11 (PA11) and polyamide 12 (PA12) (Figure 6c). In the first research, microstructures and characteristics of TPS/PA11 polymeric systems were investigated. The proposed formulations were modified, applying a compatibilization procedure by considering carboxymethyl cellulose (CMC) in an amount of 1 g CMC/100 g dry polymeric matrix. The presence of sodium neutralized anionic groups allowed the interaction between CMC and PA11, perhaps by hydrogen bonding of the amide groups and metal complexation. The polymeric blends showed interesting mechanical characteristics (high tensile strength and high modulus), even if TPS was the major element. In the second work, the authors analyzed the compatibilization of TPS/PA12 blends by the presence of poly(ethylene-co-butyl acrylate-co-maleic anhydride) terpolymer (Lotader 3410) and bisphenol A diglycidyl ether (DGEBA). The crystallization phenomenon of PA12 in TPS/PA12 polymeric blends was determined to be deeply dependent on the DGEBA amount; however, no effect was observed for the presence of Lotader 3410 t. 

Moreover, Tureèková et al. [200] analyzed the preparation of polymeric-based systems composed by an aromatic-aliphatic copolyester, having wheat starch and 35 mol % of aromatic ester units, plasticized by 15, 20, or 30 wt % of glycerol. Blends prepared from copolyester and native starch, at ratios of 55:45, 65:35, or 75:25 by weight, were analyzed; tensile strength values varied between 9 and 30 MPa. Deformation at break of the polymeric blends was very low, while wettability characterization of the analyzed systems would not reduce possible utilization as package products. 

In the case of polystyrene, Tomy et al. [201] investigated the characteristics of oxidized and native corn starch/polystyrene-based systems under reactive extrusion, using zinc octanoate as a catalyst. The systems were realized by reactive extrusion, and then processed by compression molding. The data undoubtedly showed that the used catalyst induced cross-linking between PS and starch, and that the induced oxidation of the starch magnified its reactivity. Systems did not show signs of swelling or antimicrobial growth. Yongjun et al. [202] realized copolymer by reactive grafting of starch with polystyrene (starch-g-PS) by using ionic liquid 1-ethyl-3 methylimidazolium acetate ([EMIM]Ac) as the solvent and potassium persulfate as the initiator. The obtained analyses showed that ionic liquid suspension of starch, before polystyrene grafting, is a useful procedure for the synthesis of amphiphilic, polysaccharide-based graft copolymers with high grafting amount. 

Only one paper is available that considered the tuned degradation of PVC when blended with TPS [203]; thanks to the extensive variety of uses for PVC, a complete comprehension of disintegration steps is of fundamental significance from an ecological viewpoint. 

Lastly, an extensive number of results are accessible on the potential use of thermoplastic starch in a blend with EVOH [204,205,206,207]. Generally, starch systems show relatively interesting barrier characteristics at low moisture levels and plasticizer amount, with respect of traditional membranes, such as EVOH (copolymer of vinyl alcohol and ethylene) or polyamide, which are frequently mentioned as control materials for oxygen barriera. The difficulty with starch membranes is to verify the appropriate stability between interesting barrier and mechanical characteristics. Commonly, great amount of plasticizer amount is chosen to gain a soft material, but in this situation the barrier characteristics are going to be obviously reduced. Even if starch shows higher diffusion to carbon dioxide and oxygen than ethylene-vinyl alcohol, it is indeed considerably economically advantageous to use it; thus, comparable or higher barrier materials, in comparison with EVOH-based multilayer formulations, can be obtained by considering greater thicknesses.

It has been proven that the blending of starch and PVA enhanced the disintegration of starch-filled renewable polymers [115,208]. In general, PVA/starch blend films show certain limitations, such as high affinity to water and weak mechanical characteristics. In particular, barrier and tensile performance decrease with higher starch amount, resulting from their partial compatibility, especially in the absence of plasticizers. Consequently, the main approaches to overcome these limitations comprise the use of chemicals (such as cross-linkers and surfactants) to modify the compatibility during blending, the use of modified PVA and starch instead of native PVA and starch, respectively, as well as the incorporation of nanofillers to improve their properties. Zhao et al. [209] modified starch to tackle this problem by using methylated corn-starch (MCS) and then blended it with PVA. The water absorption capacity of PVA/MCS decreased by a factor of two when compared with that of PVA/native starch. On the other hand, Jayasekara et al. [210] modified surface compatibility of PVA/starch films with the aid of chitosan since the surface roughness of PVA was lower than that of starch. However, PVA/starch blend surfaces had an intermediate roughness between those of individual PVA and starch, which remained unchanged with the addition of chitosan. 

### 5.2. Chitosan

Because of the large applications of chitosan in various fields, blends with synthetic polymers, having a wide range of physicochemical properties, have been prepared in various occasions, with solution blending investigated by many workers [211,212]. Polyvinyl alcohol and polyethylene are among the synthetic polymers that have been frequently blended with chitosan. 

The combination of good mechanical properties and hydrophilicity of PVA with the biological activity of chitosan offers a good opportunity to produce beneficial blend films with high antimicrobial effects, high formability, good strength, and high barrier proprieties, despite the fact that the elongation at break may be a limiting factor for packaging applications [213]. 

The progress of scientific investigations on polyethylene/chitosan composites has gained significant consideration, especially those prepared by melt processing, because of their superior control of the final material’s properties with respect of solvent evaporation methods. The result of the work by Lima et al. [214] specified that chitosan tunes the viscosity, loss modulus, storage and torque modulus (i.e., melt viscosity), and the mixture chitosan/PE-g-MA compatibilizer has a comparable, even if negligible, consequence. In the case of higher fillers amounts, (more than 15 wt %) the PE-g-MA influenced the rheological behavior of the mixtures, maybe improving matrix–filler interactions and working as an active compatibilizer [215].

The selection of the polymers to be blended with the chitosan depends on the property to be conferred or boosted. For example, the affinity to water characteristic of chitosan is changed by blending with polymeric matrices, such as PEG and PVA. Chitosan was similarly blended with a great number of polymeric matrices, such as polyamides, pol (acrylic acid), gelatin, silk fibroin, and cellulose to improve mechanical characteristics. 

Chitosan/nylon 11 blends, at different ratios, were produced [216] and results revealed that the physical properties of nylon 11 were greatly affected by the addition of chitosan in the blended films and that good biodegradability of the resulting blends was observed. Smitha et al. [217] have considered the characterization of crosslinked blends of chitosan/nylon 66 at different weight compositions. The obtained results showed good indication for dehydration of dioxane and moderate water sorption (50–90%) of the blends, with no significant effects on the mechanical stability of the blends. 

High temperature melting PET was also tested in combination with chitosan [218]. The PET/Chitosan blends showed a noteworthy antibacterial characteristic towards Gram-positive and Gram-negative bacteria, which improved at higher content of chitosan films. In addition, tensile strength and deformation at break of PET/chitosan films reduced with increasing chitosan amount. Consequently, the investigations of PET/chitosan systems underlined the possibility to utilize the proposed systems in the food industry as antimicrobial packages.

Mascarenhas et al. [219] blended chitosan with polystyrene to enhance mechanical and physical characteristics of chitosan, and to improve its functionality towards some specific applications: The versatility of the blends, such as film-forming ability, hydrophilicity, biodegradability, and biocompatibility are comparable with the existing blends.

Carrasco-Guigón et al. [220] realized PP/chitosan-based composites, applying the extrusion procedure and using chitosan at 9% (*w*/*w*) and PP-g-MA at 5% (*w*/*w*) as a compatibilizer. The presence of chitosan increased the wettability of the films, thanks to its high affinity to the water, while crystallinity and mechanical characteristics of PP reduced with the presence of chitosan (this behaviour could be predictable, due to the limited mechanical performance of chitosan bio-based polymeric matrix). In addition, due to the typical antimicrobial activity of chitosan, this material can be used to develop food containers to increase the conservation of food for a longer time in respect to traditional polymeric matrices.

Fernandez-Saiz et al. [221] reported, for the first time, about water barrier blends of chitosan with EVOH copolymers by solution casting: Optimal properties in terms of microstructure, optical characteristics, biocide, and water barrier activity could theoretically support the development of new bio-coatings based on chitosan salts and EVOH. The chitosan/copolymer blend can preserve the transparency and the dimensional stability, even in the presence of humidity, of the neat EVOH, but showing improved water barrier and exceptional biocide characteristics when compared to chitosan.

Polyvinyl chloride (PVC)/chitosan packaging has been also investigated [146]: Ouattara et al. [222] realized chitosan-based antibacterial packages. The diffusion of acetic or propionic acid from thin films (44–54 mm) was analyzed after water immersion at various pH (5.7, 6.4, or 7.0) and temperatures (4, 10, or 24 °C). Due to the antimicrobial characteristics of chitosan, these polymeric blends can be used as antibacterial packages [223]. Nevertheless, chitosan-based films may absorb water and swell on extended time contact. Therefore, the checking of geometric features, such as porosity, is recommended.

### 5.3. Cellulose 

Cellulose, which is crystalline and an insoluble material in water, becomes appropriate for the realization of thin systems if it is transformed in cellophane [126]. Other than cellophane, ethyl, hydroxyl-ethyl, cellulose acetate, and hydroxyl-ethyl cellulose are commercially-available treated celluloses with good toughness, transparency, flexibility, and resistance to fats and oils [224]. Cellulose acetate can be used in combination with other bio-based polymers. Suvorova et al. [225,226] reported about the use of cellulose diacetate (CDA) mixed with potato or corn starch: Enhancements in barrier and mechanical properties were found when methyl cellulose was compounded with starch-whey protein, other polysaccharides, or lipids [227,228]. A recent study aimed to identify polycaprolactone as a candidate for blending with methyl cellulose, which can be synergistically employed in a layered system. PCL significantly lowers the water vapor permeability and increases the puncture resistance when compared to methyl cellulose (7.3 × 10^−11^ gm/m^2^s Pa) [229].

Examples of CA in combination with petroleum based polymers can be found in blends or laminated PET, even if usage today is much reduced with the availability of the lower cost biaxially oriented polypropylene (BOPP) [230], while the use of PVC combined with CA is strictly related to ultrafiltration purposes [231], and few studies can be found where authors considered the blending as a way of aiming to reduce the amount of (PVC) waste products in the environment and to increase their biodegradability [232,233]. Its combination with polyamide is even restricted to membrane applications [234].

Because CA is expandable in a similar way to polystyrene, there are available studies in which surface roughness and foam morphology of cellulose acetate sheets have been compared with PS [235], and a few papers are present on their blending [236,237]. 

### 5.4. PHB 

Neat PHB shows several drawbacks, such as a high degree of crystallinity and thermal instable behavior. Consequently, its blending with petrochemical-based polymeric matrices showing low degradation values, but superior mechanical characteristics can be an answer to gain environmental and eco-sustainable advantages. The blending approach of PHB and PET might guarantee variations in the performance of the produced disposals, such as the improved mechanical characteristics of the PET or PHB biodegradability (Figure 6d) [191]. Dias et al. [238] noted that comparable temperatures during the melting phenomenon were detected for PHB/PET blends without any important interactions between them. Numerous works regarding the investigation of the main characteristics of LDPE/PHB blends have been carried out and are reported in the literature [239,240]. There is confirmation that LDPE/PHB blends are not miscible matrices with fine distribution of phase boundaries between dispersed phase and matrix. In accordance to Pankova et al. [241], the morphology of these blends showed that the minor constituent (PHB) forms band-like fibrils entrenched in the LDPE polymer. If the content of PHB is higher than 16 wt %, the blend system undertakes microstructure modification from oriented PHB structure to isotropic one, where the PHB fibrils convert into a network. The dissimilarity between the two microstructures reveals the dissimilar values of water permeability of the produced systems. Consequently, the amount of PHB in the blends adjusts the microstructure and, as a result, determines the water barrier characteristics. The same effect was found in the case of PP/PHB blends [242], characterized by a reduction of stiffness and crystallinity in the presence of a PHB polymer, and at the same time the blend showed an increase of flexibility in relation to the content of PP in the blend. Olkhov et al. [243] investigated the replacement of ether functional groups belonging to PHB, having reduced affinity to water with more hydrophilic groups (amide) belonging to polyamide, while Abdelwahab et al. studied how to advance the compatibility of PHB and PS, which have analogous processing temperatures but are basically incompatible, by using commercial compatibilizing ingredients based on PS random copolymers, containing methyl methacrylate or maleic anhydride comonomers [192] (Figure 6e). PHB/ Polyvinyl acetate (PVAc) or PVA blends were widely analyzed: PVAc and its modification, as the PVA, are miscible with PHB in the melting region. In the case of PHB/PVAc (74/26) blend, a pronounced enhancement in the deformation at break, related to the reduction of crystallinity values of the blend, was noted, determined by the presence of PVAc. Other than this, PVAc addition inhibited the second crystallization phenomenon of PHB at room temperature and; therefore, the blends demonstrated unchanging physical characteristics during storage at room temperature [244].

### 5.5. PLA 

Data provided by the literature include evaluation of properties of melt-blended PET containing small amounts of PLA, or blends containing greater amounts of PLA obtained by solution casting: These polymeric blends are characterized by reduced mechanical properties in relationship to the high content of PLA in PET, making it unappealing for industrial use [245]. The main reasons for these reductions can be found in: (a) PET processing temperature (∼260–300 °C), well over the melting temperature of PLA (∼160 °C), causing the PLA degradation and chain scission during blend compounding; and (b) no miscibility of the two polymeric matrices, which has been observed even for blends containing only small contents of PLA (5 wt %) [246]. The different polarity is also in charge of the limited compatibility of PLA with polyolefins; therefore, it is common to add a compatibilizer, such as polyethylene-grafted maleic anhydride (PE-g-MAH), in order to increase the characteristics of blends [247]. Alternatively, taking into account difficulties that need to be overcome in processing, modification by selecting a less expensive polymer, like PVC, is quite limited, so PLA, and a few numbers of articles concerning PVC/PLA-based blends have been published. It was proved that PLA stabilized the thermal degradation of PVC; however, a better compatibility was found only when MAH was considered in the blend (phase separation disappeared in the presence of MAH and the formation of MAH-g-PVC was confirmed by the increase of glass transition temperature of PVC) [248].

In the case of PS-containing blends, it is well recognized that polystyrene possessed limited degradation and narrow affinity to the water, which made the disposal of PS difficult after use. In order to modify the degradation process of PS, PS was blended with TPS, realized using several plasticizers, and the results indicated that blending of PLA with PS might be one of the best ways to find an equilibrium between cost effective PLA and also find reduced properties of PS [249,250]. Imre et al. [193] found a correlation between structure and interactions (Figure 6f), by determination of dispersed particles size, calculation of the Flory–Huggins interaction solubility parameters, and by the quantitative estimation of the composition dependence of tensile strength. 

In addition to the identification of a possible process for refining of polylactide (PLA) toughness, polyamide (PA) with elevated toughness and strength was utilized to realize PLA/PA blends [251]. There is a limited number of works proposed in the literature that considered a PLA blend with polyamides, and the system result is still not totally understood [252]. Stoclet et al. [253] analyzed the microstructure and mechanical characteristics of this system and assumed that PLA/PA11 is a low interfacial tension blend with quite good compatibility, while Dong et al. [254] analyzed the result of introducing ethylene glycidyl methacrylate-graft-styrene-co-acrylonitrile (EGMA-g-AS) rubber particles in PLA or PA11 phases on the mechanical characteristics of PLA/PA11 blends. They detected a 78- and 5.2-fold enlargement in deformation at break and impact strength, respectively, in ternary blends, in which EGMA-g-AS is mainly dispersed in the PLA phase. They also concluded that the presence of EGMA-g-AS did not modify the dispersion of polymeric phase microstructure of PLA/PA11 blends. In the proposed works, several aspects of the microstructure, interfacial and coalescence characteristics of PLA/PA11 continue to be uncertain or contradictory. The possibility of having a bio-based PA has been also evaluated [255,256], where it was confirmed that low molecular weight epoxy resin could have a substantial role as a reactive compatibilizer in PLA/PA 610 blends.

Notwithstanding its good thermal and mechanical properties, the highly hydrophobic nature of PLA leads to low hydrolytic degradation rates. In general, hydrophobicity means the poor ability for holding-up water. As water uptake is an essential step to degradation process, PLA is often blended with synthetic biopolymers, such as PVA, in order to enhance its biodegradability. Li et al. [257] recommended that the blending of PVA with PLA could lead to promising ecofriendly materials for packaging applications with high performance, such as good mechanical properties and thermoplasticity. Furthermore, Shuai et al. [258] stated that poly(L-lactide) was immiscible with PVA with completely different Tg peaks being observed from differential scanning calorimetry (DSC) results, which is ascribed to the absence of hydrogen bonding in amorphous regions.

### 5.6. PBS 

PBS is a costly polymer with limited mechanical properties, consequently it is not good for final use. Because polyethylene terephthalate is a material that is hardly degradable by microorganisms, the possible blending of PET with PBS could enhance the disintegrability of a non-degradable matrix in the blend. Threepopnatkul et al. [259] found that PET/PBS blends were totally immiscible. At the same time, an overall decrease of tensile properties, such as strength, modulus, and elongation at break, in the presence of PBS, in comparison with neat PET thin film, was measured. In the case of blending with polyolefins, due to the fact that HDPE has comparable mechanical properties with PBS, the blending of these two matrices not only modified the cost but also improved the overall mechanical performance of the final blend. Aontee et al. [172] found that a HDPE and PBS blend was immiscible, showing many different morphologies depending on HDPE content (from spherical domain to worm-like and elongated structure, up to coalescence;, moreover, it was observed that yielding and breaking strength gradually decreased with increasing HDPE content. The use of PVC was recently considered by Chuayjuljit et al. [260], who designed how to recover the stiffness and induce degradability of poly(vinyl chloride) by inserting poly(butylene succinate) (PBS) in the blends: The obtained blends (from 10 to 50 wt % of PBS phase) showed an increase in the impact strength, elongation at break, and inclination to biodegrade when compared to the neat PVC, only with increasing content of the biodegradable PBS phase. 

### 5.7. PBAT 

Polybutylene adipate-co-terephthalate (PBAT) has great deformation at break comparable to a thermally processable elastomer, a characteristic that should be considered in case of blending with PET, having interesting thermal and mechanical characteristics, low gas diffusion, chemical resistance, and good transparency, but limited degradation in presence of water and microbials. As shown by Thongsonget al. [261], with increasing content of PBAT, thermal resistance of PBAT/PET film was decreased in comparison with neat PET film. In parallel, the increase of PBAT would result in lower values for elastic modulus and tensile strength, while the deformation at break would considerably improve, particularly when the PBAT amount is above 40 wt %. Nevertheless, the limited presence of 10 wt % of PBAT could even enhance deformation at break and tensile strength, to give values more elevated than neat PET system.

### 5.8. Bio-PE-Based Blends

A limited number of papers discussed the blending of bio-based polyolefins, and only one example was found with bio-based PLA. As reported in Brito et al. [262], the authors investigated the effect of ethylene-glycidyl methacrylate (E-GMA) and ethylene-methyl acrylate-glycidyl methacrylate (EMA-GMA) copolymers as compatibilizer agents in PLA/Bio-PE blend. The data underlined that the use of the E-GMA and EMA-GMA copolymers significantly enhanced the impact strength of the PLA/Bio-PE blend, thanks to the reaction between hydroxyl or carboxyl groups in PLA and the epoxy groups in the copolymer matrices.

## 6. Nanocomposites of Hybrid Blends and Bio-Based Nanofillers

Generally, the functional characteristics of bio-based polymers in relation to their physical and functional characteristics are required to be modified to accommodate foodstuff necessities, applying diverse strategies and modifications, as chemical or physical changes (crosslinking) or blending with different polymers and or fillers/components (compatibilizers or plasticizers). Consequently, several works have been made to design polymeric-based materials, extracted from renewable sources, with similar properties to those of traditional ones, with the final scope of reducing the environmental problems induced by the use of plastics [263,264]. The properties to be considered are numerous and may incorporate water vapor and gas diffusivity into polymeric chains, mechanical characteristics, thermal processability, sealing capability, resistance (chemical attacks (acid), grease, water, UV light, etc.), machinability (on the packaging line), antifogging ability, optical characteristics (transparency), printability, and economic aspects. It is significant to understand that no single natural material will assure all possible business areas and utilization. Consequently, an increasing attention is seen in the development of packages involving multilayer systems, in analogy to the conservative approach to create multilayer high-barrier films (e.g., a bio-based laminate implying plasticized chitosan or a starch based system combined with PHA or PLA), the final substance offers the barrier characteristics to gases and mechanical strength similar to a laminate with an external layer of polyamide (PA) or ethylene-vinyl alcohol (EVOH) assembled with LDPE [265]. Therefore, the characteristics of any biopolymer are analyzed focusing the attention on the main fundamental characteristics:

(a) Gas barrier properties: It was studied that biodegradable polymeric matrices show fine resistance to the oxygen transmission and, presently, several strategies are achieved to adjust their barrier characteristics. Nevertheless, the oxygen transmission rate (OTR) is superior in bio packaging with respect to conventionally-utilized polymeric matrices, with the consequence that the shelf-life of foods decreased. Similarly, the CO_2_ barrier performance of polymeric matrices is also largely significant in packages for fresh foodstuffs. Conventional high barrier systems contain the presence of numerous layers to create a system with the necessary features; similarly, two or more bio-based polymeric matrices can be arranged to design a polymeric system taking the essential requirements [266].

(b) Water vapor barrier characteristics: The main restrictions of bio-based packages are their hydrophilic performance. Even if water vapor barrier performances of bio-polymeric matrices are related with conventional matrices, a composite biopolymer can be designed and innovated with transmittance rates rather similar to the conventional plastics. The biodegradable package is a high-quality alternative for products that necessitate high water vapor diffusion. Deep study has been performed to modulate the water diffusion characteristics of bio-systems also realizing numerous layers or developing nanocomposite approach [267].

(c) Light barrier characteristics: The sunlight energy accelerates the corrosion and deterioration processes that unfavorably influence photosensitive foods. Sunlight, in addition, operates as a catalyst to enhance the rancidity of lipids. Additionally, light provokes the oxidative modification in the polymeric, resulting in the deterioration of polymeric materials, counting both bio-based and conventional packages. To prevent the photo degradation of polymeric matrices and foodstuffs, UV stabilizer and absorber components can be included in the package systems [268]. 

(d) Compostability: It is one of the main significant conditions of bio polymeric matrices, as it changes their attitude to the disintegration and modifies the degradation behavior of wastes into advantageous soil products. The disintegration of polymeric materials in composting soil depends essentially on characteristics of the polymeric sources. The first stage of disintegration in composting soil is typically based on the hydrolytic process that damages the polymeric materials, and hydrolytic level is related to the water vapor diffusion of the substance [269,270,271].

It has been recommended that intrinsic weaknesses of bio-based packages, such as reduced barrier characteristics, hydrophilicity, low heat deflection temperatures, restricted processing gap, and low mechanical performance, may be overcome by a nanocomposite approach [140,272]. The potential of layered clay mineral nanoparticles has been established, thanks to their commercial accessibility, relatively high barrier and mechanical performance enhancements, low price, and moderately simple processability. Nevertheless, it may be underlined that although nanoclays are largely available natural sources, they are not biodegradable or renewable. Several other nanoparticles, comprising nano-metal oxide or metal have been utilized to enhance the matrix thermal performance and add some functions, such as strong antibacterial action.

In this scenario, bio-based nanofillers, obtained from no totally crystalline bio-polymeric matrices, such as cellulose, chitin, or starch, or from amorphous lignin, have recently obtained significant attention owing to their elevated accessibility and low density. Nevertheless, while literature on fully biodegradable nanocomposite blends containing nanofillers from plant and animal origin is quite extensive, research on hybrid systems is relatively unexplored. According to this, a deep revision of current results and overall performance of hybrid (bio-based plus synthetic matrices) polymeric nanocomposites containing different nanofillers (mainly bio-based ones) will follow.

### 6.1. Starch Based Hybrid Nanocomposites

Naderizadeha et al. [273] realized starch-based films by blending with PVA, produced by solvent casting. Two nanofillers (sodium montmorillonite (Na-MMT) and SNCs (starch nanocrystals)) were used and their achievements on overall performance of nanocomposites were investigated. This study unfolded the synergistic influence of SNC and Na-MMT to modulate mechanical characteristics, mostly in 3 wt % of the total amount of nanofillers in 50:50 (*w*/*w*).

To enhance the mechanical and moisture diffusion characteristics of starch-based systems, attention has been directed to produce nanocomposites by adding nanoscale particles [274]. For example, previous studies have revealed that whatever the clay type, the presence of nanoclays into PVA/starch, PCL/starch [275,276], PE/starch [277], and PP/starch blends [278] determined an enhancement in the material stiffness, mechanical characteristics, water resistance, and thermal stability. Tang and co-workers [279] developed several PVA–starch/nano-silicon dioxide (SiO_2_) biodegradable blend films and discovered that the mechanism responsible for the improved tensile strength and water resistance of the composites was the formation of a strong chemical bond between nano-SiO_2_ and PVA–starch blend. They also analyzed the biodegradability of nano-SiO_2_ reinforced starch/PVOH nanocomposites and the results underlined that nanoparticles had no important control on biodegradability of films. Spiridon et al. [280], on the other hand, analyzed degradation of clay/starch/PVOH nanocomposites and determined that films biodegradation is related to both typology and number of nanoparticles, and the nanoparticles delayed the biodegradation degree.

The PVA/starch/clay nanocomposite as a food packaging material has been also developed [281] and, in active packaging, some additives have been also included in the formulation to promote an antimicrobial or bacterial adhesion inhibitory effect [282]. Quite recently, Tang and Alavi [283] reviewed the use of starch in combination with PVOH and established that thermally-processable starch/PVOH/montmorillonite micro and nanocomposites could demonstrate intercalated and exfoliated structures throughout the extrusion process technique.

### 6.2. Cellulose-Based Hybrid Nanocomposites 

Carboxymethyl cellulose (CMC) added to polyvinyl alcohol (PVA) biopolymers have been extensively utilized for advanced biodegradable films in the packaging sector: CMC are compatible and miscible bio-based matrices, due to the existence of multifunctional groups on polymeric chains; as a result the blend strategy of these matrices can facilitate the production of bio-based matrices with characteristics that consent their use in the promising field of bio-packages. PVA/CMC blend can be utilized as a novel bio-blend matrix to develop and realize bio-based nanocomposite systems with improved characteristics. El Achaby et al. [284] established that the presence of 5 wt % CNC (cellulose nanocrystals) in PVA/CMC improved the tensile modulus and strength by 141% and 83%, respectively, and the water vapor diffusivity was weakened by 87%. Moreover, the developed systems reinforced with filler at thenanoscale level preserved similar transparency of the PVA/CMC system (transparency level in the visible region was estimated at around ∼90%), highlighting that the CNC were well distributed at the nanoscale. The proposed nanocomposites showed interesting adhesion characteristics and the great number of functional groups present in the CNC’s surface and in the macromolecular chains of the PVA/CMC system are useful to enhance the interfacial relationship between the polymeric blend and CNC. As a result, these eco-friendly organized bio-nanocomposite systems with higher characteristics are estimated to be of practical use in the food packaging sector. By using MMT, Taghizadeh et al. [285] analyzed that MMT content drastically influenced the rate of starch solubilization and explained the reduction of the degradation rate in MMT/PVA-/CMC. Additionally, at 5% (*w*/*w*) of MMT, the systems are characterized by the lowest water absorption capability (WAC) % levels.

### 6.3. Chitosan-Based Hybrid Nanocomposites

Cano and co-authors [45] designed and studied the combination of cellulose nanocrystals (CNC) in polymeric blend of poly(vinyl alcohol) (PVA) and pea starch (2:1 content) blend films, to enhance the physical and functional characteristics and the stability of produced formulations throughout storage, reducing some negative aspects of starch-based films. Specific amounts (1, 3, and 5 wt %) of CNC were selected to modulate the barrier properties of PVA/pea starch based film used as control. No changes in water vapor permeability (WVP) were registered adding the different content of CNC; this behavior can be related to the enhancement in the hydrophilic nature of the films, as also observed by the overall migration levels in polar and non-polar food simulants. The nanocomposites are characterized by lightly rigid and more stretchable behavior, crystallization phenomenon of PVA was moderately reduced by CNC addition [45].

**Figure 7:**
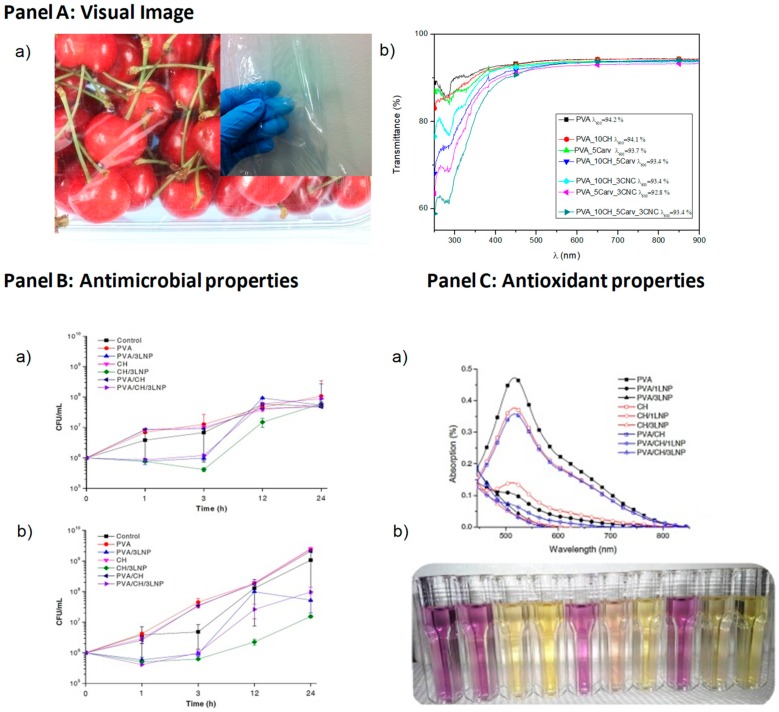
Panel **A**: Illustration of PVA/10CH/5Carv/3CNC formulation (**a**); and UV-Vis curves of PVA formulations (**b**) [286]. Panel **B**: Antibacterial study of PVA, PVA/CH, and PVA/CH/LNP nanocomposites, on the increase of plant pathogenic bacteria *Pectobacterium carotovorum* subsp. *odoriferum* (Pco) (CFBP 1115) 1 × 10^6^ CFU/mL (**a**); and *Xanthomonas arboricola* pv. *pruni* (Xap) (CFBP 3894) 1 × 10^6^ CFU/mL (**b**) [48]. Panel **C**: Antioxidant properties migrating substances of PVA/CH/LNP nanocomposites immersed in methanol solution for 24 h, measuring the absorbance level at 517 nm (**a**); and colorimetric deviation of the DPPH methanolic solution (**b**) [48].

Multifunctional poly(vinyl alcohol) (PVA) and (10 wt %) chitosan (CH) films reinforced with (3 wt %) CNC obtained from kiwi *Actinidia deliciosa* lignocellulosic wastes, obtained after the pruning were produced, and combined with (5 wt %) carvacrol, used as the active ingredient, for prospective industrial application [286]. Luzi and co-author developed and studied novel PVA and PVA/10CH systems with modulate characteristics of main significance in thefood packaging sector [286]. The microstructure analysis highlighted that no changes in the PVA and PVA/CH cross section areas were found due to the addition of nanocellulose (CNC) and/or carvacrol (Carv), underlining the positive synergism among the ingredients. The authors studied the optical and colorimetric characteristics, the data highlighted that no changes were detected on transmittance level and colorimetric appearance of PVA and PVA/CH blend, which were kept also in the case of four components-based formulations (Figure 7, Panel A). The different produced PVA-based formulations combined with carvacrol showed an important antioxidant effect, whilst the presence of carvacrol and chitosan induced an antimicrobial effect [286]. Yang and co-authors analyzed the effect of introducing lignin nanofillers (LNP) at two different weight ratios (1 and 3 wt %) on chitosan/polyvinyl alcohol (PVA) hybrid nanocomposite films, realized by using a solvent casting technique [48]. Antibacterial studies (Figure 7, Panel B) revealed the capability to decrease the microbial growth of Gram-negative bacteria, suggesting the possibility of using these films against the growth of microbial plant/fruit pathogens in the food package sector. Additionally, the results from the presence and combined effect of CH and LNP in the antioxidation property (Figure 7, Panel C) predicted their use not only in the food packaging sector, but also in the biomedical sector (drug delivery, tissue engineering, wound healing), where new antiseptic innovations are frequently necessary [48]. 

Another application of chitosan hybrid nanocomposites was analyzed by Sadeghi and co-authors [287]. In that work, ethylene vinyl alcohol copolymer (EVOH) and chitosan were combined with nano zinc oxide (nano-ZnO) and glycerol used as plasticizer, these systems were deeply analyzed and developed for food packaging systems. The functional characteristics of EVOH nanocomposites underlined improved barrier, mechanical, and transparency characteristics. The antiseptic characteristics were enhanced by adding chitosan. The addition of plasticizer determined a reduction of barrier properties and, at the same time, an increase of deformation at break (ε_b_) was detected. The addition of nano-ZnO improved barrier, mechanical, and antibacterial characteristics [287].

### 6.4. Polyester-Based Hybrid Nanocomposites

Nuñez et al. [288] focused the investigation on the possible efficiency of sepiolite clay on the final performance (tensile toughening) of matrices in ternary blending systems, having PLA as the polymeric phase and low-density PE as the distributed phase. Results highlighted that the blends realized without clay are characterized by high thermal stability and tensile durability, compared to those realized with sepiolite. The nanocomposite blends showed comparable thermal profile, lower tensile strength and Young’s modulus values, and enhanced deformation at break and tensile toughness with respect of PLA nanocomposites. These data evidenced how clay dispersion, type of microstructure of the various blends, localization of the sepiolite in several phases, thermomechanical degradation of the PLA through melt blending, and grafting level of the utilized compatibilizer ingredients were critical for the successful performance of the materials. As’habi et al. [289] considered the blending of poly lactic acid (PLA)/linear low-density polyethylene (LLDPE) polymeric nanocomposites, realized using two commercial-grade nanoclays: due to the selective localization of the nanoclays in the PLA phase, the systems that were realized applying a two stages mixing procedure showed interesting biodegradability, microstructure, and improved melt resistance in comparison to the one step mixing process.

## 7. Production, Market, and Future Perspective of Hybrid Bio-Based Polymers 

**Figure 8:**
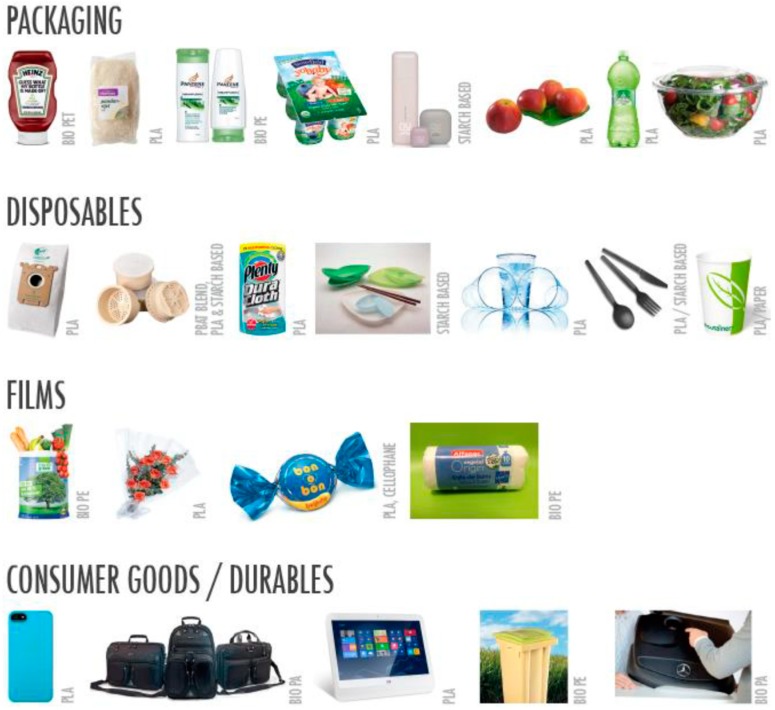
Bio-based plastic products.

Every year, 125 million tons of plastic materials are utilized worldwide, of which 25% is utilized for packaging aims. It is obvious that the possible business for packaging systems is massive if they can be realized with good functionality, processability, and at an interesting and advantageous price. This is the high request for the producers of biopolymers oriented to the packaging sector, that have to deal with conventional plastics, which are available at low price, are easy to be realized, and with modulated and improved packaging characteristics [290]. Table 4 summarizes a list of foodstuffs, their barrier necessities, the classic packaging systems and green selections and, at the same time, with their technology readiness levels; while in Figure 8, examples of commercial bio-based plastics and polymers for packaging and disposals are provided.

The large amount of green food packaging systems are quite expensive in respect to fossil-based systems [291]. The price for commodity plastics is largely centered in the range $1.32–$3.3/kg. Unluckily, no exact evaluation of price for traditional and bio-based systems is accessible. It was estimated that bio-based materials are three to five times more expensive in comparison to traditional packaging systems [292]. The higher drivers of the cost to realize green materials include the cost for mobilizing biomass wastes, cost for technical and scientific innovations, and the lack of economies-of-scale (introduction of a eco-friendly bio-based creation chain, from natural wastes and biomass wastes to final bio-based material is a difficult and expensive procedure, moreover novelties are required to adjust present bioconversion procedures to new typologies of feedstock, or improve new procedures).

**Figure 9:**
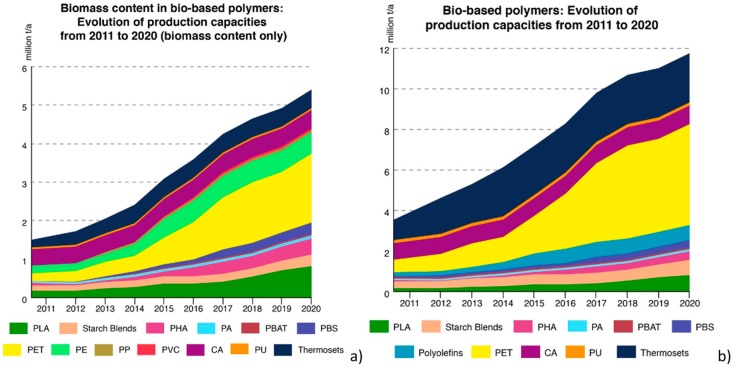
Bio-based polymers: Biomass content utilized in bio-based polymeric matrices (**a**); and development of production capacities from 2011 to 2020 (**b**) [282].

The “Bio-Based Polymers Producer Database,“ which is incessantly updated by the Nova-Institute, exhibits that Europe’s running situation in creating bio-based polymeric matrices is restricted to a few number of polymeric matrices. The European community has so far determined a solid role, principally in the area of starch blending materials (polymeric blending based on the combination of polymers with starch or thermoplastic starch) and it is anticipated to continue to be solid in this specific sector for the following few years. Commercial examples of starch-based blends with conventional polymers (Linear low-density polyethylene (LLDPE), polypropylene (PP), High Impact Polystyrene (HIPS)) can be found, such as Cereplast and Teknor Apex products (Starch/LLDPE, Starch/PP, Starch/HIPS from 30/70 up to 50/50 wt %, Starch/LLDPE and Starch/PP up to 50/50 wt %), TPS/synthetic copolyesters/additives from BIOP and TPS/polyolefins hybrid from Biograde and Cardia Bioplastics. Similarly, for polybutylene terephthalate (PBT), new progresses in the realization of bio-based 1,4 butanediol (BDO) have established that the ecofriendly course to the polymer is commercially operable and its realization is deliberated to be introduced by 2020. The market size is quite differentiated, taking into account that some sectors taken by conventional plastics (PS, PVC, PET) have been now targeted by eco-friendly compostable plastic materials, such as poly lactic acid (PLA), polybutylene succinate (PBS), and polyhydroxyalkanoates (PHA’s). Exact objective markets contain the catering-service manufacturing in single uses, such as plates, disposable eating utensils, foamed cups, containers, and bowls (Figure 9).

Other markets are represented by green or moderately bio-based non-biodegradable thermally-processable materials, such as eco-friendly PVC, PP, PE, or PET: In 2010, the inclusion of a 100% bio-material comparable to HDPE by Braskem changed the bioplastics sector, requiring at the same time a renewable resource component and a compostability behavior. Obtained from sugar cane ethanol, this material is chemically comparable to petrochemical extracts, thus can be utilized in the same sector. Similarly, bio-PVC can be realized totally from natural sources. Since 2007, Solvay Indupa, the Brazilian arm of Belgium-based chemical colossal Solvay, established strategies to utilize Brazilian sugarcane ethanol as a PVC raw material. At the moment, Bio-PET contains bio-based glycol extracted from sugar cane ethanol and the amount of renewable carbon was estimated at around 20%. 

The main markets for this creation are ketchup bottles (Heinz), soda bottles (Coca Cola), and Pantene shampoo packages (Proctor and Gamble). Nevertheless, the price of bio ethylene glycol is yet significantly superior in respect to its traditional equivalent based one, and so market diffusion is limited. Even if nylon 6 and nylon 6,6, based on renewable components, are not currently presented on the market area, Rennovia has established its process to realize both hexamethylene diamine and adipic acid monomers to facilitate bio nylon 6,6 and nylon 6 [294].

On the other hand, even though it is obvious that important development has been completed in reducing numerous characteristic issues with aliphatic polyesters, there is still a requirement for further advancements. Almost all the processes that are practicable nowadays utilize, also, traditional petrochemical and non-compostable ingredients, or do not present the development economics needed (the content of these ingredients that can be utilized are also restricted if compostability is yet a condition). In detail, in some blanket positive assessment of bio-based and biodegradable plastics, it is frequently forgotten that energy from petroleum fuels is also utilized in their fabrication—be it in the sowing of crops, harvesting, fermentation, transport, etc. It is, consequently, necessary to evaluate a product’s complete life cycle, because only after this evaluation is it feasible to carry out scientifically sound life cycle assessment comparisons and reach a logical conclusion about a product’s sustainability.

Finally, sustainability of the polymeric matrix production will center on the successful presence of novel polymeric matrices that are extracted/obtained from yearly renewable sources. These resources must have reasonable end of life possibilities (e.g., they should be able to be reused by chemical or physical means, biodegraded to inoffensive mixtures, or minimally destroyed to recuperate the energy amount). Alternatively, “unsustainable polymers” may yet contribute as key roles in advancing sustainability, as lately underlined in a research work in Chemical and Engineering News [283], centered on food packaging systems with whole multilayered polymeric systems, where it has been conclusively remarked how the cost of plastic packaging is certainly high, but the cost of not using it may be higher. 

## 8. Conclusions

Biopolymers, considered as green polymeric matrices or plastics realized from natural feedstock by synthetic routes, frequently have poorer characteristics and performances in respect to traditional polymeric matrices. One route to be monitored for achieving characteristic combinations necessary for the packaging sector is their blending, in the presence or not of nanosized fillers. The present review underlined how hybrid blends containing renewable polymers, in concurrence with synthetic polymers and additives, have great potential in enhancing the moisture and gas barrier properties of bio-based materials, and how they are not economically practicable to be utilized without polymeric blends with low-priced plastics of comparable necessary characteristics. On the other hand, the overall performance of polymeric blends is undoubtedly correlated to blend compositions and phase morphologies that need to be optimized by using compatibilization methods or a nanocomposite approach. In order to consider their potentials and enter new markets, other than the packaging sector where a feeble interest has already risen, scientific research should strongly focus its efforts on extending their use and improving general performance in other parallel or different sectors.

## Figures and Tables

**Table 1:** Physical properties of polymers applied in packaging and food packaging.

Polymer	Packaging Types	Thermal		Mechanical			Permeability		
		T_g_ (°C)	T_m_ (°C)	T (MPa)	ε_B_ (%)	OP	H_2_O: WVTR (g/m^2^/day)	O_2_ (g/m^2^/day)	CO_2_ (ml μm m^−2^ day^−1^ atm^−1^)
**PET**	Bottles, microwaveable and ovenable trays, boil in-the-bag products	70–87	243–268	48–72	20–300	++	15–20	100–150	300–600
**HDPE**	Jars and other rigid containers, pallets, films, or layers for dry food	−125 to −90	135	22–31	100 ≥ 1000	+++	7–10	1600–2000	12,000–14,000
**PVC**	Wrapping films, bottles, trays, containers	60–100	n.d.	40–51	40–75	++	0.5–1.0	2–4	400–10,000
**LDPE**	Films (wrapping, carrier bags, pouches), bottles	−125 to −100	112–135	8–31	200–900	++	10–20	6500–8500	20,000–40,000
**PP**	Cups and containers for frozen and microwaveable food, lids, thin-walled containers (yoghurt)	−10	167–177	31–41	100–600	+	10–12	3500–4500	10,000–14,000
**PS**	Disposable cups, plates and trays, boxes (egg cartons), rigid containers (yoghurt)	100	n.d	35–51	1–4	++		4500–6000	14,000–30,000
**PA**	flexible packaging of perishable food, such as cheese and meat	50–60	220	40–52	5–10	++	300–400	50–75	n.d
**PVA**	Films for moisture barrier, confectionery products	70–75	215–220	25–30	220–250	++	n.d.	n.d.	n.d
**EVOH**	Thin films for dry/fatty food, multilayer	60–65	180–150	45–110	180–250	+++	1000	0.5	n.d

Tg—glass transition temperature; Tm—melting temperature; T—tensile strength; ε_B_—elongation at break (%); OP—overall optical properties including haze, gloss, and transmission of visible light; permeability (H_2_O, water vapor; O_2_, oxygen gas; CO_2_, carbon dioxide gas) for polymeric films.

Oxygen permeability and water vapor transmission rate were evaluated (WVTR) in (g/m^2^/24 h) in tropical conditions (90% Relative Humidity (RH) at 38 °C).

n.d.: not defined. +: low. ++: medium. +++: high. Polyethylene terephthalate (PET); high density polyethylene (HDPE); polyvinyl chloride (PVC); low density polyethylene (LDPE); polypropylene (PP); polystyrene (PS); polyamide (PA); poly (vinyl alcohol) (PVA); poly (vinyl alcohol-co-ethylene) (EVOH).

Partially reprinted from: [42,105,108,109,112,118,119,120,121,122].

**Table 2:** Bioplastics as food contact materials [129].

Bioplastic	Main Food Applications
**Starch-based polymers**	Substitute for polystyrene (PS). Used in food packaging, disposable tableware and cutlery, coffee machine capsules, bottles.
**Cellulose-based polymers**	Low water vapor barrier, poor mechanical properties, bad processability, brittleness (pure cellulosic polymer), Regulated under 2007/42/EC. Coated, compostable cellulose films. Used in the packaging of bread, fruits, meat, dried products, etc.
**Polylactide (PLA)**	Possible alternative of low- and high-density polyethylene (LDPE and HDPE), polystyrene (PS), and poly terephthalate (PET). Transparent, rigid containers, bags, jars, films.
**Polyhydroxyalkanoates (PHA)**	Family of many, chemically different polymers Brittleness, stiffness, thermal instability.
**Bio-based polypropylene (PP) and polyethylene (PE)**	Mainly based on sugar cane. Identical physicochemical properties.
**Partially bio-based (PET)**	Alternative to conventional PET. Up to 30% bio-based raw materials. Used in bottles.
**Bio-based polyethylene furanoate (PEF)**	Better barrier function than PET. Up to 100% bio-based raw materials. May be used in the future in bottles, fibers, films.
**Aliphatic (co)polyesters**	Includes polybutylene succinate (PBS), polyethylene succinate (PES), and polyethylene adipate (PEA). Used in disposable cutlery.
**Aliphatic-aromatic (co)polyesters**	Includes polybutylene adipate terephthalate (PBAT), polybutylene, and succinate terephthalate (PBST). Used as fast food disposable packaging, PBAT for plastic films.
**Polycaprolactone (PCL)**	Biodegradable polyester. Low melting temperature, easily biodegradable. Used in medical applications.
**Polyvinyl alcohol (PVOH)**	Used for coatings, adhesives, and as additive in paper and board production.

**Table 3:** Characteristics and food use of polysaccharides extracted from animals and vegetables [140].

Polysaccharide	Properties	Main Food Applications
Starch 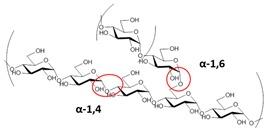	Biodegradable Transparent Odorless and tasteless Retrogradation high elongation and tensile strength	Flexible packaging: Extruded bags Nets for fresh fruit and vegetables Rigid packaging Thermoformed trays and containers for packaging fresh food.
Cellulose 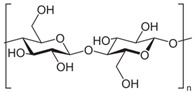	Biodegradable Good mechanical properties Transparent Highly sensitive to water Resistance to fats and oils Need to perform modification, use of plasticizer, or polymer blend	Cellophane membranes.
Chitin 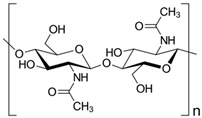	Biodegradable Antibacterial and fungistatic properties Biocompatible and non-toxic Highly transparent	Coffee capsules Food bags Packaging films
Chitosan 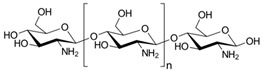	Biodegradable Biocompatible and non-toxic Antifungal and antibacterial properties Good mechanical properties Barrier to gases High water vapor permeability Brittle—need to use plasticizer	Edible membranes and coatings (strawberries, cherries, mango, guava, among others) Packaging membranes for vegetables and fruit

**Table 4:** Barrier properties of selected foodstuffs with classic and bio-based packaging systems [293].

Packed Product	Barrier Requirements	Classic Packaging Solution	Bio-Based Packaging Solution	Technology Readiness Level
Meat/fish	High barrier against oxygen and gas (aroma);	Trays (PS, PP, PVC with EVOH + LDPE or PVC as coating) + foil (PVC) or lid, bags, for short term storage; waxed paper (wrapping), paperboard external packaging; transparent films (PP, PE)	Multilayer packaging materials, functional bio-based coating (modified starches) + antimicrobial and anti-fogging systems	On the market (as pilot packaging on selected markets); still more expensive than conventional solutions
Fresh cheese	High barrier properties; grease, water, O_2_, CO_2_ and N_2_, aroma and light. MAP (80% N_2_, 20% CO_2_)	Transparent films/foils; bags (e.g., LDPE/ EVA /PVdC /EVA), trays, wrapping films (PE, laminated), plastic cups (HDPE, PP, PS) + high barrier lid (PA/LDPE)	Eco-paper for short term storage (wrapping); PHA/modified PLA films	On the market, still more expensive than conventional plastics
Dairy products/liq uids	High barrier properties; water vapor (scavenging moisture), O_2_, light high/moderate for grease and aroma	Waxed paper, LDPE, PVC, or aluminum-coated/laminated paper or paperboard, plastic films (BOPP), metal cans	Paper/paperboard coated with bio-based materials	Close to market
Salad (flexible packaging)	High oxygen barrier, water resistant	Transparent laminated PP films	PLA films (perforated) Coated paper with bio-based films + transparent window	On the market, still more expensive than conventional plastics
Fruits/vegetables	Medium barrier properties (water vapor)	Perforated PP, OPP, LDPE; PVC films/bags, trays, pouches, overwraps; PS/PP trays	Molding pulp—trays PLA films (perforated) Edible coatings (polysaccharides: xanthan gum, starch, cellulose, HPC, MC, CMC, proteins: chitosan, corn zein, wheat gluten) + low barrier packaging films	On the market (molded pulp trays); on the market (PLA as pilot packaging in selected markets, e.g., for tomatoes); still more expensive than conventional solutions
Take-away food	Grease, thermal insulation	Polystyrene foam trays	Paperboard with grease barrier coating on the inside	On the market

BOPP—biaxially oriented polypropylene, CMC/carboxymethyl cellulose, EVA = ethylene vinyl acetate, EVOH = ethylene vinyl alcohol, HDPE = high-density polyethylene, HIPS = high-impact polystyrene, HPC = hydroxypropyl cellulose, LDPE = low-density polyethylene, MC = methyl cellulose, OPP = oriented polypropylene, PA = polyamide, PE = polyethylene, PET = polyethylene terephthalate, PHA = polyhydroxyalkanoate, PLA = poly lactic acid, PP = polypropylene, PS = polystyrene, PVC = polyvinyl chloride, PVdC = polyvinylidene chloride.
